# Light‐Responsive Nanoantennas Integrated into Nanoscale Metal–Organic Frameworks for Photothermal Drug Delivery

**DOI:** 10.1002/smsc.202400088

**Published:** 2024-05-10

**Authors:** Manuela Cedrún‐Morales, Manuel Ceballos, Enrica Soprano, Giulia Zampini, Ester Polo, Beatriz Pelaz, Pablo del Pino

**Affiliations:** ^1^ Departamento de Física de Partículas Centro Singular de Investigación en Química Biolóxica e Materiais Moleculares (CiQUS) Universidade de Santiago de Compostela 15705 Santiago de Compostela Spain; ^2^ Centro Singular de Investigación en Química Biolóxica e Materiais Moleculares (CiQUS) Universidade de Santiago de Compostela 15705 Santiago de Compostela Spain; ^3^ Departamento de Bioquímica y Biología Molecular Centro Singular de Investigación en Química Biolóxica e Materiais Moleculares (CiQUS) Universidade de Santiago de Compostela 15705 Santiago de Compostela Spain; ^4^ Departamento de Química Inorgánica Centro Singular de Investigación en Química Biolóxica e Materiais Moleculares (CiQUS) Universidade de Santiago de Compostela 15705 Santiago de Compostela Spain

**Keywords:** controlled drug release, metal organic–frameworks, nanocomposites, photothermal therapy, plasmonic nanoparticles

## Abstract

Nanoscale metal–organic frameworks (NMOFs) exhibit unique properties for drug delivery, including ultrahigh storage capabilities, biocompatibility, biodegradability, and sustained release of encapsulated cargo. However, due to their localized electronic states, MOFs are nonresponsive to external stimuli such as light or magnetic fields. This study investigates the integration of light‐responsive nanoantennas into NMOFs to enhance their application as smart drug delivery nanosystems. By integrating gold bipyramid nanoantennas within ZIF‐8 and NU‐1000 NMOFs, core@shell nanosystems are created with photothermal capabilities. Utilizing cresyl violet as a model drug, the loading and release dynamics of these nanosystems are analyzed, demonstrating controlled drug release under near‐infrared (NIR) light stimulation. Photothermal release studies conducted in living cells reveal the potential of these nanocomposites for spatiotemporal targeted, light‐activated drug delivery. Further evaluation of the NU‐1000 nanocomposite loaded with chemotherapeutics—doxorubicin, carboplatin, and oxaliplatin—in both 2D and 3D cell cultures shows the nanosystem effectiveness in cell internalization and therapeutic NIR activation. The findings demonstrate that the incorporation of stimuli‐responsive elements into NMOFs offers a promising approach for developing advanced drug delivery platforms.

## Graphical abstract

Core@shell nanocomposites, with plasmonic nanocores encased in metal–organic frameworks, are explored for their potential in near‐infrared‐responsive drug delivery. These nanocomposites are engineered with drug‐locking polymer coatings to enhance cargo‐loading and feature photothermal properties for controlled drug release. Efficacy is demonstrated in 2D and 3D cell culture models, highlighting their promise in targeted cancer therapy among other nanomedicine applications.

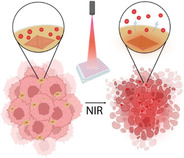

## 1 Introduction

The pursuit of precision medicine—an approach where treatments are personalized based on an individual's genetic profile and environmental interactions—is increasingly becoming a tangible goal, propelled by breakthroughs in drug delivery technologies. Central to this progress is the innovative use of nanotechnology, particularly through the development of advanced nanocarriers, which promises to direct therapeutic agents to the site of pathology with unparalleled precision.^[^ ^1^ ^]^ The evolution of such advanced nanomedicines is set to transform treatment paradigms, making them not only more effective but also minimally invasive, thus offering fresh optimism in addressing longstanding healthcare challenges.^[^ ^2^ ^]^

Among the various innovations in the field of drug delivery, the concept of controlled release stands out as a transformative approach. It aims to precisely manage the rate and location of drug administration, enhancing therapeutic effectiveness by maintaining optimal drug levels at the target site for the required duration.^[^ ^3^ ^]^ This approach is crucial in reducing side effects and improving patient compliance, especially in treatments like chemotherapy where the margin between effective and toxic doses is narrow.

The evolution of nanocarriers, including liposomes,^[^ ^4^ ^]^ solid lipid nanoparticles (NPs),^[^ ^5^ ^]^ dendrimers,^[^ ^6^ ^]^ polymeric,^[^ ^7^ ^]^ and/or inorganic NPs,^[^ ^8^ ^]^ has been instrumental in advancing targeted therapy. Engineered to navigate the body's complex biological landscape, these nanoscale vehicles are designed to evade immune detection and accurately release their therapeutic payload directly at the site of the disease. The integration of these carriers with stimuli‐responsive mechanisms marks a substantial advancement in drug delivery technology.^[^ ^2^, ^9^ ^]^ The employment of near‐infrared (NIR) light for noninvasive targeting, alongside other stimuli like ultrasound, alternating magnetic fields, and uncaging chemistry, expands the horizon of therapeutic possibilities.^[^ ^10^ ^]^ These methods offer various mechanisms—mechanical, thermal, and chemical—for precise drug activation and release, showcasing the versatility and sophistication of smart drug delivery systems (DDSs) in meeting diverse therapeutic needs with high precision and efficiency.^[^ ^11^ ^]^

The rationale for choosing NIR light, specifically within the optical biological windows (650–1350 nm), is strategic for its deep tissue penetration and minimal absorption by water and hemoglobin, facilitating targeted intervention with reduced risk to surrounding nontargeted tissue. Recent studies underscore the potential of NIR light in enhancing the efficacy of DDSs.^[^ ^12^, ^13^ ^]^ Similarly, significant advances on porous plasmonic nanosystems highlight the integration of thermoplasmonic effects with NIR light for remote delivery and/or activation of drugs in living cells.^[^ ^14^, ^15^, ^16^ ^]^

Nanoscale metal–organic frameworks (NMOFs) serve as versatile platforms with remarkable porosities, allowing for the encapsulation of a diverse range of molecules.^[^ ^17^, ^18^ ^]^ However, the development of precision DDSs generally benefits of precise regulation of drug release to avoid lack of effectivity and undesirable side effects.^[^ ^11^ ^]^ Stimuli‐responsive NMOFs represent a promising solution to solve these problems, integrating the outstanding properties of these materials with the stimuli‐controlled delivery of the encapsulated cargo.^[^ ^2^, ^19^ ^]^

The strategic combination of light‐responsive agents with NMOFs allows the development of DDSs capable of controlled cargo release through conformational changes or photothermal conversion under illumination. Hence, the creation of new core–shell nanocomposites (NCs), featuring an Au NP core within an NMOF, gives rise to a synergistic effect between both components, presenting an excellent candidate for an advanced DDS.^[^ ^15^, ^19^, ^20^ ^]^

ZIF‐8 and NU‐1000 stand out as two main examples of widely utilized NMOFs in biomedical applications.^[^ ^16^, ^21^, ^22^ ^]^ However, certain drawbacks associated with these particles hinder their effectiveness as DDSs, particularly due to structural fragility in biological fluids leading to uncontrolled early release of the encapsulated drug.^[^ ^21^, ^23^, ^24^ ^]^

**Figure 1:**
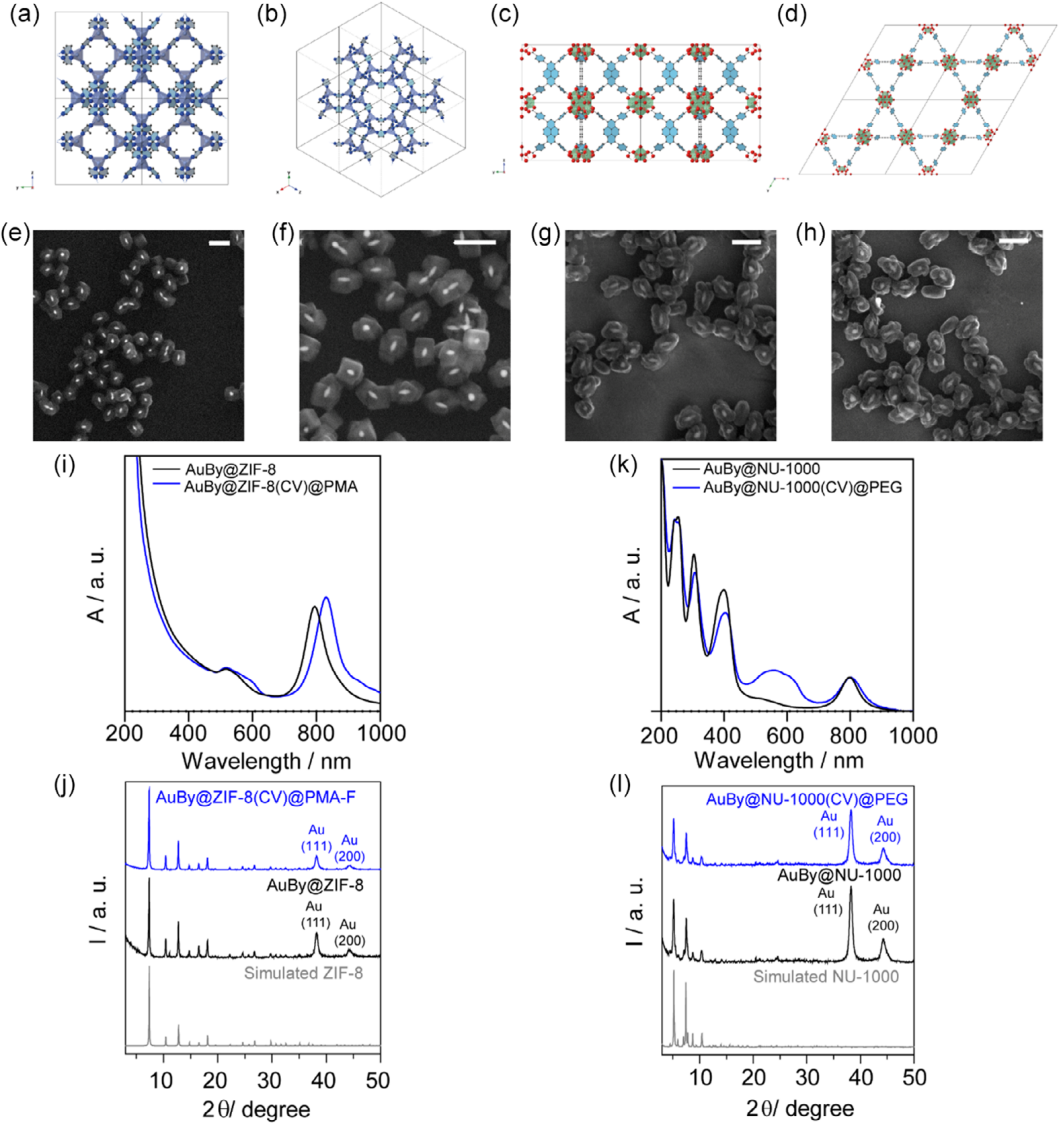
ZIF‐8 and NU‐1000 structures: a) from the *x*‐axis and b) the [110] plane for ZIF‐8; c) from the *a*‐axis and d) the *z*‐axis for NU‐1000. FE‐SEM images of e) AuBy@ZIF‐8, f) AuBy@ZIF‐8(CV)@PMA, g) AuBy@NU‐1000, and h) AuBy@NU‐1000(CV)@PEG. i) UV–vis absorption spectra and j) PXRD diffractograms for ZIF‐8‐based NCs and k) UV–vis absorption spectra and l) PXRD diffractograms for NU‐1000‐based NCs.

Hence, in this study, we propose the design of microporous plasmonic nanosystems based on ZIF‐8 (**Figure** **1**a,b,e,f) and NU‐1000 (Figure 1c,d,g,h), which serve as nanoplatforms for encapsulating specific cargo with controlled triggered release employing NIR as external stimulus. Building upon previous work demonstrating enhanced stability in different NMOF structures after surface modification using polymers, ZIF‐8 and NU‐1000 NMOFs were selected for investigating their potential as NIR‐controlled DDSs. The proposed NCs feature a plasmonic core, which consists of a light‐responsive gold nanobipyramid (AuBy) that is encapsulated by a NMOF, creating a core–shell NC structure. These systems effectively combine the optical properties of the AuBy with the porosity and protective features of the NMOF shell. The NMOF shell serves as a support to load and retain the selected cargo molecule, while the NIR‐responsive plasmonic core facilitates selective release upon resonant plasmonic excitation and photothermal heating.

## 2 Results and Discussion

### 2.1 NCs Synthesis and Characterization

AuBys were selected as the core of the system. Due to their specific asymmetric geometry, AuBys can exhibit enhanced light absorption per NP compared to other shapes. Additionally, by adjusting the quantity of seeds in the synthesis, we can easily fine‐tune localized surface plasmon resonance (LSPR) to align with the desired wavelength. AuBys were synthesized following a previously reported method,^[^ ^25^ ^]^ utilizing pentatwinned seeds that facilitated the crystallization of pentagonal bipyramids with dimensions of 85.0 ± 5.6 nm in length and 30.9 ± 3.0 nm in width (Figure S1a–d, Supporting Information), which due to their aspect ratio possess a LSPR at around 810 nm (with slight variations due to the dispersion media, Figure S1e, Supporting Information).

The synthesis of AuBy@ZIF‐8 NCs was carried out using a method that included multiple water washing steps and the subsequent redispersion of AuBys in hexadecyltrimethylammonium bromide (CTAB) with a concentration of 0.16 mg mL^−1^, as outlined in prior studies.^[^ ^15^, ^16^, ^26^ ^]^ This surfactant facilitates the growth of ZIF‐8 onto the surface of AuBy but does not support the growth of NU‐1000, owing to differing synthesis requirements.

Figure 1e shows field emission scanning electron microscopy images (FE‐SEM) of AuBy@ZIF‐8 NCs with a cubic shape, each featuring one AuBy per NCs with a size around 160 ± 18 nm (Figure S2a, Supporting Information). This configuration arises from a seed growth crystallization mechanism that enables the formation of a polycrystalline ZIF‐8 shell, facilitated by the presence of CTAB molecules on the AuBy's surface.^[^ ^27^ ^]^ In Figure 1f and S3, Supporting Information, FE‐SEM images of AuBy@ZIF‐8 NCs loaded with cresyl violet (CV) and coated with fluorescein‐labeled poly(isobutylene‐alt‐maleic anhydride)‐graft‐dodecyl (hereinafter referred to as PMA) to enhance stability in water^[^ ^15^, ^16^, ^26^ ^]^ reveal that the size and morphology of these functionalized NCs are consistent with those of the nonfunctionalized counterparts. This step is critical due to the rapid degradation of ZIF‐8 when exposed to CO_2_ dissolved in water, a process that typically unfolds within minutes.^[^ ^28^ ^]^ Notably, the ZIF‐8 within the PMA‐coated NCs remained stable and did not convert into ZnCO_3_, in contrast to the degradation observed in the non‐PMA‐coated NCs, as shown in Figure S4a–d (Supporting Information). Further comparison with the PMA‐coated NCs (AuBy@ZIF‐8(CV)@PMA NCs in Figure S4e–f, Supporting Information) confirms their enhanced stability in water, maintaining integrity for a minimum of 7 days.

In the case of AuBy@NU‐1000 NCs, the synthetic protocol presents additional challenges owing to the specific conditions required to facilitate NU‐1000 growth. These conditions involve the use of organic solvents such as *N*,*N*‐dimethylformamide (DMF), monocarboxylic acids like acetic acid (HOAc) as modulators, and high temperatures (≥90 °C). The primary challenge was to identify an appropriate capping agent that would enable the transfer of AuBys into DMF media. Based on previous reports within our group,^[^ ^19^ ^]^ we opted for a polyethylene glycol linker HS‐PEG‐OMe (2 kDa). After PEGylation, the AuBys were redispersed in DMF and utilized as seeds in the synthesis of AuBy@NU‐1000 NCs. The PEG chains are aimed to attract Zr moieties, initiating the MOF‐shell growth.^[^ ^29^ ^]^ This process resulted in NCs with an average length of ≈170 ± 16 nm, as illustrated in Figure 1g and S2b, Supporting Information. To provide colloidal stability and prevent particle agglomeration, AuBy@NU‐1000 NCs underwent functionalization with mPEG‐PO_3_ after being loaded with CV (Figure 1h and S5, Supporting Information).^[^ ^30^ ^]^ NC's PEGylation preserved their morphology for at least 7 days (Figure S6, Supporting Information).

Figure 1i displays the absorption spectra of AuBy@ZIF‐8 NCs both in MeOH and for the CV‐loaded, PMA‐coated variant. An observed redshift in the LSPR absorption band for AuBy near 800 nm can be ascribed to three main factors: 1) the transition of the surrounding media from MeOH to water, 2) the incorporation of CV, evident around 600 nm (Figure S7, Supporting Information), and 3) the effect of PMA functionalization. This functionalization alters the dielectric environment surrounding the NCs, consequently leading to the observed redshift in LSPR absorption.^[^ ^31^, ^32^ ^]^

Figure 1j presents powder X‐ray diffraction (PXRD) diffractograms for AuBy@ZIF‐8 NCs and its PMA‐coated variant, demonstrating the structural stability of the NCs after PMA coating. The patterns align with the simulated PXRD pattern of ZIF‐8, with the first two Au reflections corresponding to the (111) and (200) crystallographic planes at 38.1° and 44.2° two‐theta degree, respectively.

Figure 1k illustrates the absorption spectra of AuBy@NU‐1000 NCs before and after CV loading and PEGylation, highlighting the presence of CV with absorption around 600 nm. Notably, no changes in the LSPR absorption band were observed.

Figure 1l displays the PXRD diffractograms alongside the simulated pattern of NU‐1000, revealing a match with the reflections of the NU‐1000 reference at low angles. Furthermore, the first two reflections of Au (111) and (200), similar to the case of AuBy@ZIF‐8, are evident. This comparison underlines the maintained crystallinity of the NU‐1000 structure, even after the processes of CV loading and PEGylation, demonstrating stability in water for at least one week (Figure S8, Supporting Information).

The quantification of CV encapsulation was performed by measuring fluorescence in the supernatants (SN) after washing steps, before and after polymer functionalization. CV encapsulated per MOF was calculated by comparing SN fluorescence to calibration curves in MeOH and water (Figure S9, Supporting Information). The ZIF‐based NC exhibited an 8 wt% CV content before the coating process, which was reduced to 6 wt% following the addition of PMA. This reduction suggests that the polymer may enter the pores, displacing the CV, consistent with expectations from previous studies.^[^ ^15^ ^]^ This wt% corresponds to an estimated value of 1.5·10^5^ CV per NC. On the other hand, the NU‐1000‐based NC demonstrated similar trends, with a precoating 13 wt% CV content and a post‐PEGylation 10 wt%, leading to 9·10^5^ CV per NC, proving the higher encapsulation capacity of the NU‐1000 related to bigger pore sizes.

The adsorption capacities of the NCs were assessed through N_2_ adsorption isotherms, as illustrated in Figure S10, Supporting Information. Figure S10a, Supporting Information shows the adsorption isotherm of ZIF‐8 NCs. The type I isotherm shape is preserved postloading with CV, albeit with reduced uptake, decreasing from 375.2 cm^3^ g^−1^ before loading to 318.7 cm^3^ g^−1^ after the loading (at 0.8 *P*/*P*_0_) (Table S1 and Figure S11–S13, Supporting Information). Prior investigations have established the inherent flexibility of ZIF‐8 structures, facilitating the opening of the window and accommodating larger molecules than its nominal window size,^[^ ^33^ ^]^ which has been reported to be about 3.4 Å.^[^ ^34^ ^]^ In the case of the PMA‐coated sample, a significant decrease in uptake to 221.4 cm^3^ g^−1^ is observed. This reduction can be ascribed to both the filling of pores with alkylic chains from PMA and the hindrance of N_2_ diffusion into the pores due to the coating, resulting in substantial loss of the microporosity region. On the other hand, AuBy@NU‐1000 exhibited a type IV isotherm, demonstrating an uptake of 593 cm^3^ g^−1^ at 0.8 *P*/*P*_0_. Upon loading with CV, there was a reduction to 532 cm^3^ g^−1^ attributed to pore occupation. Subsequent PEGylation led to a further decrease in uptake to 172 cm^3^ g^−1^, indicating the infiltration of PEG‐PO_3_ molecules into the NU‐1000 structure, causing a near‐complete loss of micropores. Brunauer–Emmett–Teller (BET) surface area values were determined using BETSI software (Figure S14–S16 and Table S2, Supporting Information), aligning with previously reported values. Notably, the contribution of nonporous gold material to the surface area was considered negligible in comparison with the porous material.

### 2.2 Colloidal Stability

**Figure 2:**
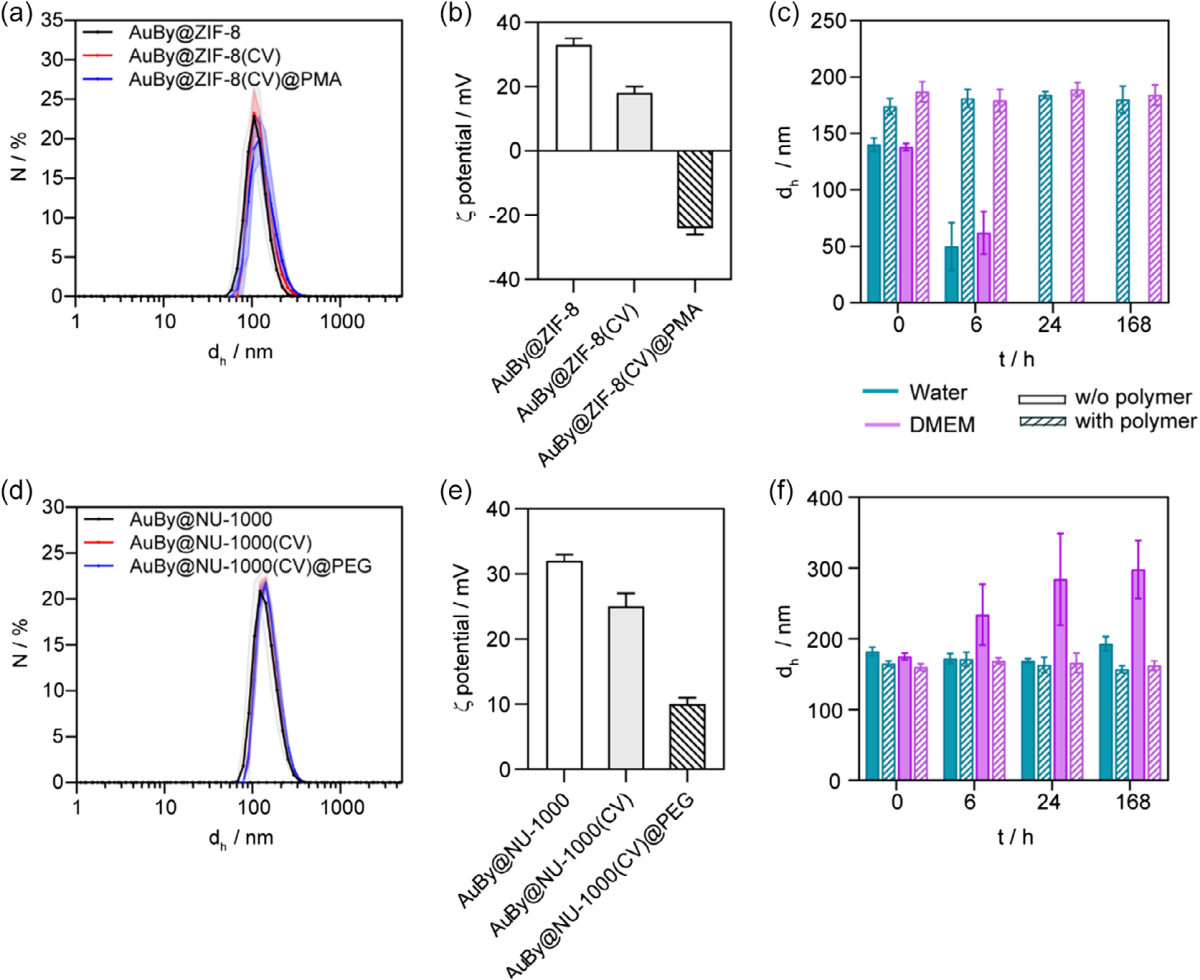
a) Hydrodynamic diameter (*d*_h_) measurements of AuBy@ZIF‐8 NCs by DLS. b) ζ‐potential of AuBy@ZIF‐8 NCs before and after loading/functionalization. c) Colloidal stability of AuBy@ZIF‐8 NCs over time in water and cDMEM media, as determined by DLS. d) *d*_h_ measurements of AuBy@NU‐1000 NCs by DLS. e) ζ‐potential of AuBy@NU‐1000 NCs before and after loading/functionalization. f) Colloidal stability of AuBy@NU‐1000 NCs over time in water and cDMEM media, as determined by DLS.

The coating of these two NCs with distinct polymers, one physically attached (PMA) and the other chemically attached (MeO‐PEG‐PO_3_), aimed to enhance their chemical and structural stability in aqueous media, along with improving colloidal stability. **Figure** **2** illustrates the colloidal characterization of AuBy@MOF NCs. In Figure 2a, dynamic light scattering (DLS) measurements of AuBy@ZIF‐8 NCs are presented before and after loading with CV and subsequent coating with PMA. The results revealed that the hydrodynamic diameter (*d*_h_) remained relatively constant at around 160 nm postloading (Table S3, Supporting Information). Following PMA coating, a slight increase in size occurred, attributed to the presence of PMA chains on the surface, featuring carboxylic acid groups capable of attracting water molecules and co‐ions, thereby marginally expanding the size.^[^ ^15^ ^]^ These results were corroborated with nanoparticle tracking analysis (NTA) measurements, allowing the determination of the size and concentration of the samples (particles·mL^−1^) (Figure S17a–c, Supporting Information). In Figure 2b, ζ‐potential measurements of the CV‐unloaded and ‐loaded ZIF‐8 NCs variants exhibit a decrease from +33.3 to +16.7 mV, respectively, possibly attributed to acetate ions from CV binding to the surface. A more significant change is observed when the NCs are coated with PMA, resulting in a ζ‐potential of −25.0 mV, indicative of free carboxylic acid groups following the hydrolysis of remaining maleic acid rings.

Figure 2c illustrates the colloidal stability of AuBy@ZIF‐8 in both water and completed cell culture media (complete Dulbecco's Modified Eagle Medium [cDMEM]), comparing samples with and without PMA coating. The uncoated sample exhibits a significant reduction in *d*_h_ almost instantly in both water and cDMEM, with a near fourfold decrease observed within 6 h. This reduction is attributed to the decomposition of ZIF‐8, a finding supported by FE‐SEM images presented in Figure S4, Supporting Information. On the contrary, the PMA coated sample shows remarkable stability, maintaining its hydrodynamic diameter over 168 h. For the NU‐1000‐based NCs, DLS measurements indicate a stable size profile post‐PEGylation around 170 nm, as depicted in Figure 2d and Table S3, Supporting Information. This consistency in size is further corroborated by NTA measurements (Figure S17d–f, Supporting Information). Furthermore, a notable decrease in ζ‐potential is observed, dropping from +31.3 to +9.9 mV, as illustrated in Figure 2e. This reduction is attributed to the negative charge of the phosphate groups, which indicates the successful PEGylation of NU‐1000. Despite these changes, colloidal stability in water remains unaffected, both with and without PEGylation. Conversely, within 6 h in cDMEM, the *d*_h_ of the NCs nearly doubles, a phenomenon likely due to the presence of salts and amino acids in cDMEM that affects the colloidal stability of NCs through electrostatic interactions, leading to aggregation. Importantly, this potential destabilization is effectively countered by PEGylation, as evidenced in Figure 2f. This aligns with the known properties of this polymer, which is widely used as a coating to enhance stability, prevent opsonization, and promote prolonged circulation in the blood.^[^ ^30^ ^]^

### 2.3 Thermal Characterization

**Figure 3:**
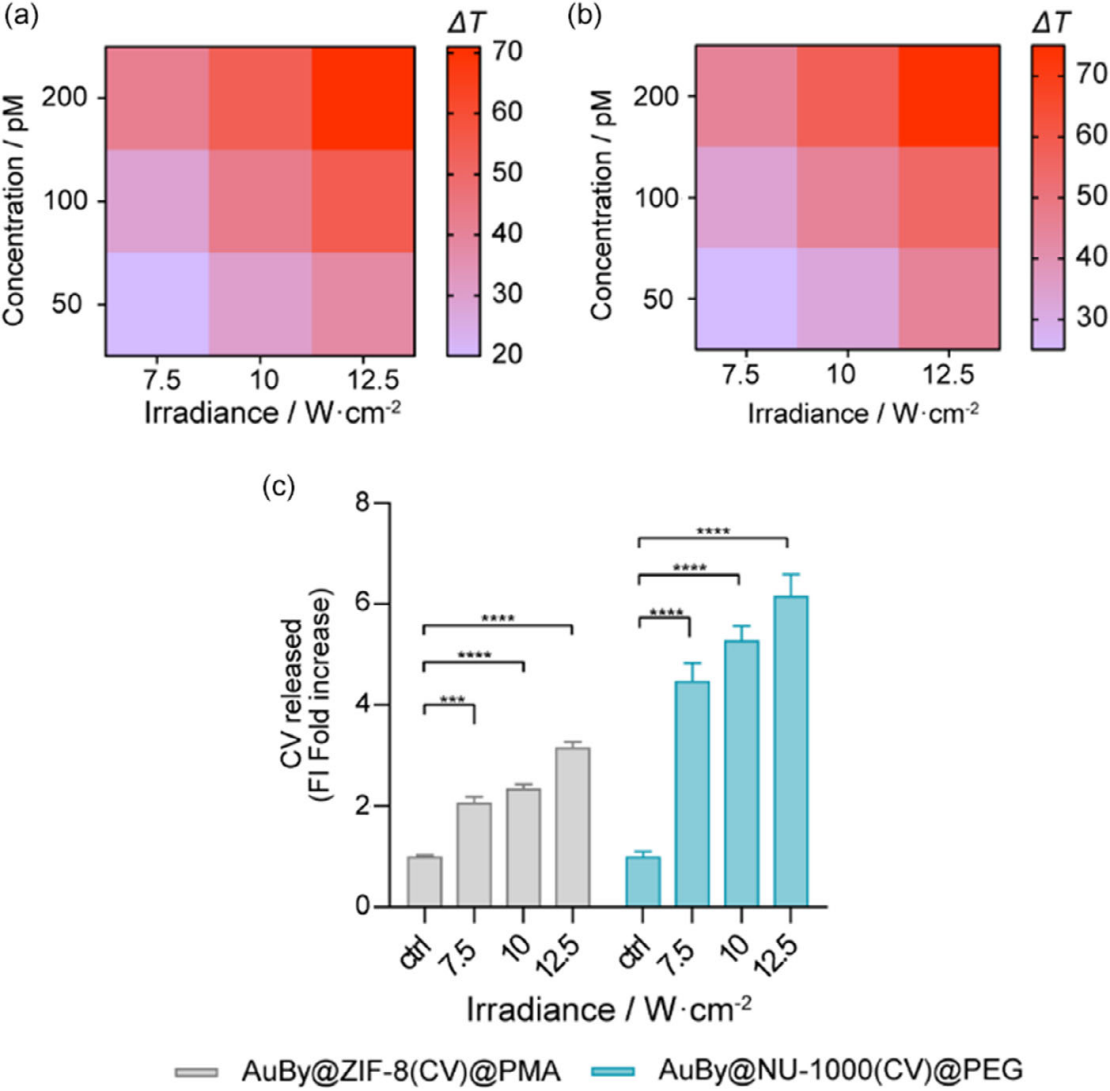
Heatmaps displaying the peak temperatures reached following 1 min of irradiation for a) AuBy@ZIF‐8@PMA NCs and b) AuBy@NU‐1000@PEG NCs. c) Fold increase FI associated with the release of CV post 1 min irradiation at a NC's concentration of 100 pM in water. All experiments were conducted in triplicate. Error bars represent standard deviation. Statistical analysis was assessed by two‐way ANOVA test (***p = 0.0002, ****p < 0.0001).

To examine the photothermal behavior of the NCs, samples were irradiated under a NIR laser (*λ* = 808 nm) matching the AuBys longitudinal LSPR, with different irradiances (7.5, 10, and 12.5 W cm^−2^), varying concentrations of NCs (50, 100, and 200 pM, as derived from NTA measurements) and various exposure times (1 and 2 min). All the irradiations were performed in a 96‐well plate using aqueous solutions containing 200 μL of each sample. The temperature *T* of the sample during each irradiation was measured using a probe to generate the dynamic *T* curves. Δ*T* irradiation time profiles for AuBy@ZIF‐8@PMA and for AuBy@NU‐1000@PEG NCs are shown in Figure S18 and S19, Supporting Information, respectively. In both cases, the heating curves show a similar trend, achieving the boiling *T* (100 °C) for the higher irradiance level (12.5 W cm^−2^) and NC concentration (200 pM). **Figure** **3**a,b presents the heatmaps correlating NC concentration with irradiance power after 1 min of exposure. As a baseline, water without NCs served as the control, exhibiting negligible temperature change, underscoring the specificity of the NC photothermal response. The heating patterns for AuBy@ZIF‐8@PMA and AuBy@NU1000@PEG NCs showed remarkable similarity, with minor deviations potentially attributable to the unique structural characteristics of each MOF type. These findings underscore the NCs’ proficiency in absorbing NIR light and converting it to thermal energy, highlighting their potential for photothermal applications.

To assess the controlled‐release performance of the CV‐loaded NC systems, we conducted irradiation tests under optimal conditions (100 pM NC concentration, 1 min exposure) using three different irradiance levels. After NIR treatment, the NCs were precipitated, allowing for the quantification of the CV released in the SN through fluorescence analysis (refer to Figure 3c and Table S4 and S5, Supporting Information for detailed data). AuBy@ZIF‐8(CV)@PMA exhibited a notable increase in CV release, achieving a threefold enhancement in fluorescence intensity (FI) relative to the nonirradiated control, highlighting the photothermal‐triggered release capability. Impressively, the AuBy@NU‐1000(CV)@PEG exhibited a sixfold FI increase in CV release at the highest irradiance compared to control, underscoring their superior responsiveness to NIR‐triggered release. Control samples, which did not undergo NIR irradiation, showed only marginal CV release, emphasizing the critical role of photothermal activation in achieving controlled drug release.

### 2.4 Thermoplasmonic Behavior Studies Inside Living Cells in 2D

**Figure 4:**
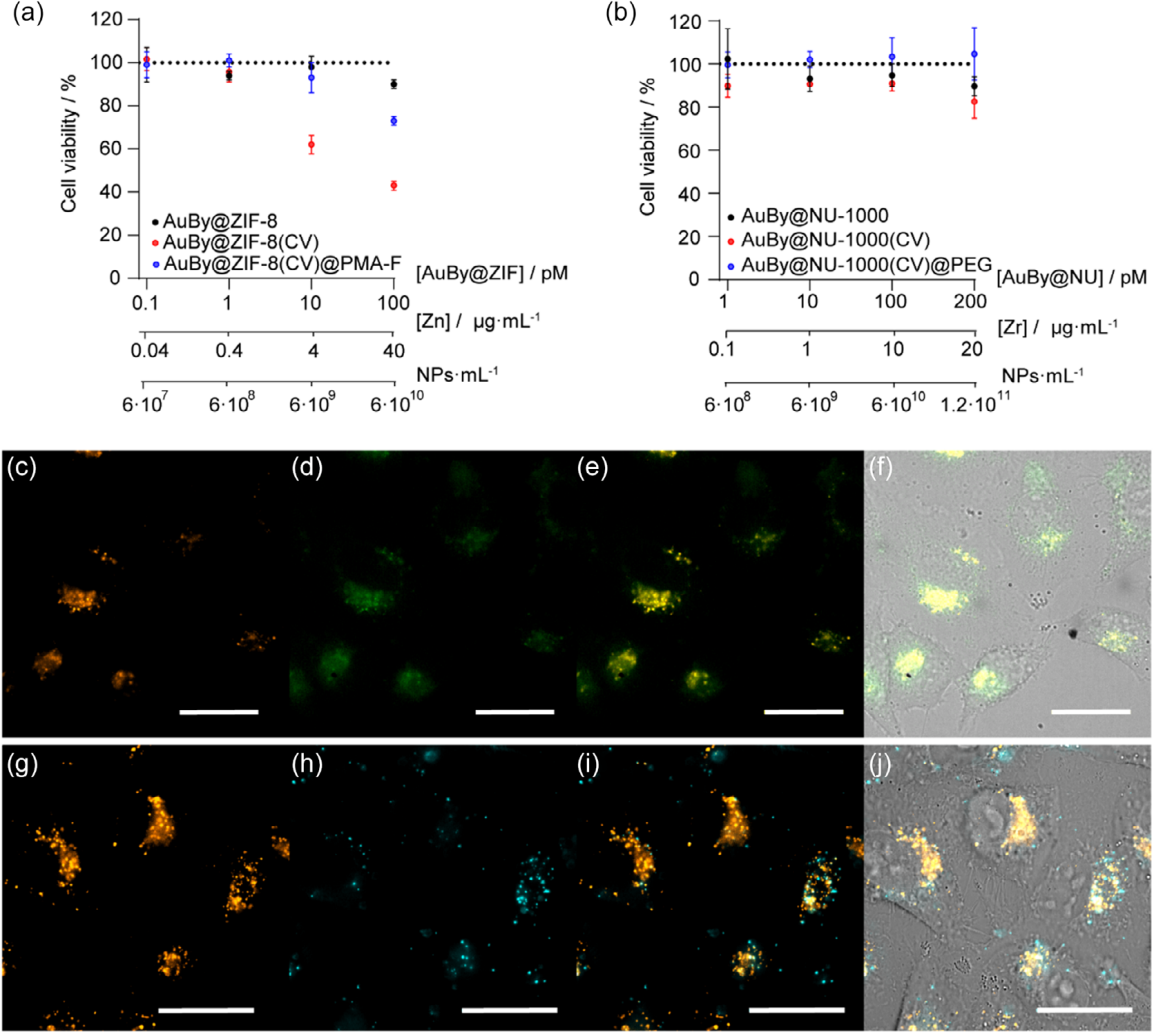
a) Cell viability of A549 cells incubated with ZIF‐8‐based NCs and b) NU‐1000‐based NCs for 24 h, assessed by MTT assay. Fluorescence microscopy images demonstrating the internalization of AuBy@ZIF‐8(CV)@PMA by A549 cells: c) CV fluorescence signal (orange), d) fluorescein fluorescence signal (green), e) overlay of CV and fluorescein signal, and f) composite image including overlay of fluorescence and brightfield channels. For AuBy@NU‐1000(CV)@PEG internalization by A549 cells: g) CV fluorescence signal (orange), h) NU‐1000 fluorescence signal (cyan), i) overlay of CV and NU‐1000, and j) composite image including overlay of fluorescence and brightfield channels. Scale bar: 25 μm.

Next, the interaction of the NCs with cells was evaluated in 2D cell cultures of A549 lung adenocarcinoma cells. The biocompatibility was assessed by determining the cellular metabolic activity using a 3‐[4,5‐dimethylthiazol‐2‐yl]‐2,5‐diphenyltetrazolium bromide (MTT) assay. This study confirmed the high viability related to both NCs at different concentrations. We used values of up to 100 pM for AuBy@ZIF‐8@PMA NCs and 200 pM for AuBy@NU‐1000@PEG NCs (corresponding to about 40 and 20 μg mL^−1^ of Zn and Zr, respectively) (**Figure** **4**a,b). AuBy@NU‐1000@PEG NCs showed no significant toxicity at any concentration after 24 h of incubation, related to the high biocompatibility of this MOF.^[^ ^35^ ^]^ On the contrary, there are notable differences observed among the ZIF‐8‐based NC samples. Bare AuBy@ZIF‐8 NCs exhibit high viability across all concentrations, attributed to the low toxicity inherent to this MOF. However, a marked increase in toxicity becomes apparent in the CV‐loaded samples before the addition of PMA, especially at concentrations exceeding 10 pM. This increase in toxicity is linked to the ZIF‐8 instability in aqueous environments, which leads to its degradation and the subsequent release of CV (Figure S20, Supporting Information). In contrast, postfunctionalization with PMA significantly enhances viability. These findings are consistent with aforementioned CV release studies, revealing that AuBy@ZIF‐8(CV)@PMA controls exhibit higher CV release percentages (≈10%) compared to AuBy@NU‐1000(CV)@PEG (≈4%) (Table S4 and S5, Supporting Information). Consequently, to prevent unspecific CV leakage and its inherent toxicity profile,^[^ ^26^ ^]^ both PMA and PEG are demonstrated to act as effective blocking agents. Nevertheless, ZIF‐8‐based NCs exhibit a marginally higher release rate, leading to increased toxicity at higher concentrations. This observation aligns with prior studies involving these coatings, confirming the efficacy and appropriateness of the selected strategy.^[^ ^15^, ^36^ ^]^

We then studied the uptake of the NCs using fluorescence microscopy. Epifluorescence microscopy imaging experiments showed the intracellular location of the NCs in the perinuclear region, as expected for the endocytic mechanisms of NCs cellular uptake (Figure 4c–j).^[^ ^17^, ^18^ ^]^ This intracellular localization indicates a clear colocalization of the CV and the NCs. For AuBy@ZIF‐8(CV)@PMA samples (Figure 4c–f), there is a clear colocalization between the CV and the PMA, whereas for AuBy@NU‐1000(CV)@PEG (Figure 4g–j), CV colocalization is confirmed using the NU‐1000 fluorescence. These findings confirm the NCs’ capability to store CV intracellularly, attributed to the effectiveness of polymer functionalization in preventing nonspecific CV leakage.

We next investigated the in vitro stimuli‐responsive release of encapsulated cargo via NIR laser (808 nm) excitation, integrated with a fluorescence microscope through a laser scanner.^[^ ^19^ ^]^ This configuration affords precise spatial and temporal irradiation control. For this experiment, A549 cells were incubated with both NCs types for 6 h. Based on prior toxicity evaluations, we opted for 100 pM of AuBy@NU‐1000(CV)@PEG and 50 pM of AuBy@ZIF‐8(CV)@PMA NCs to warrant nontoxic levels while ensuring optimal CV detection.

**Figure 5:**
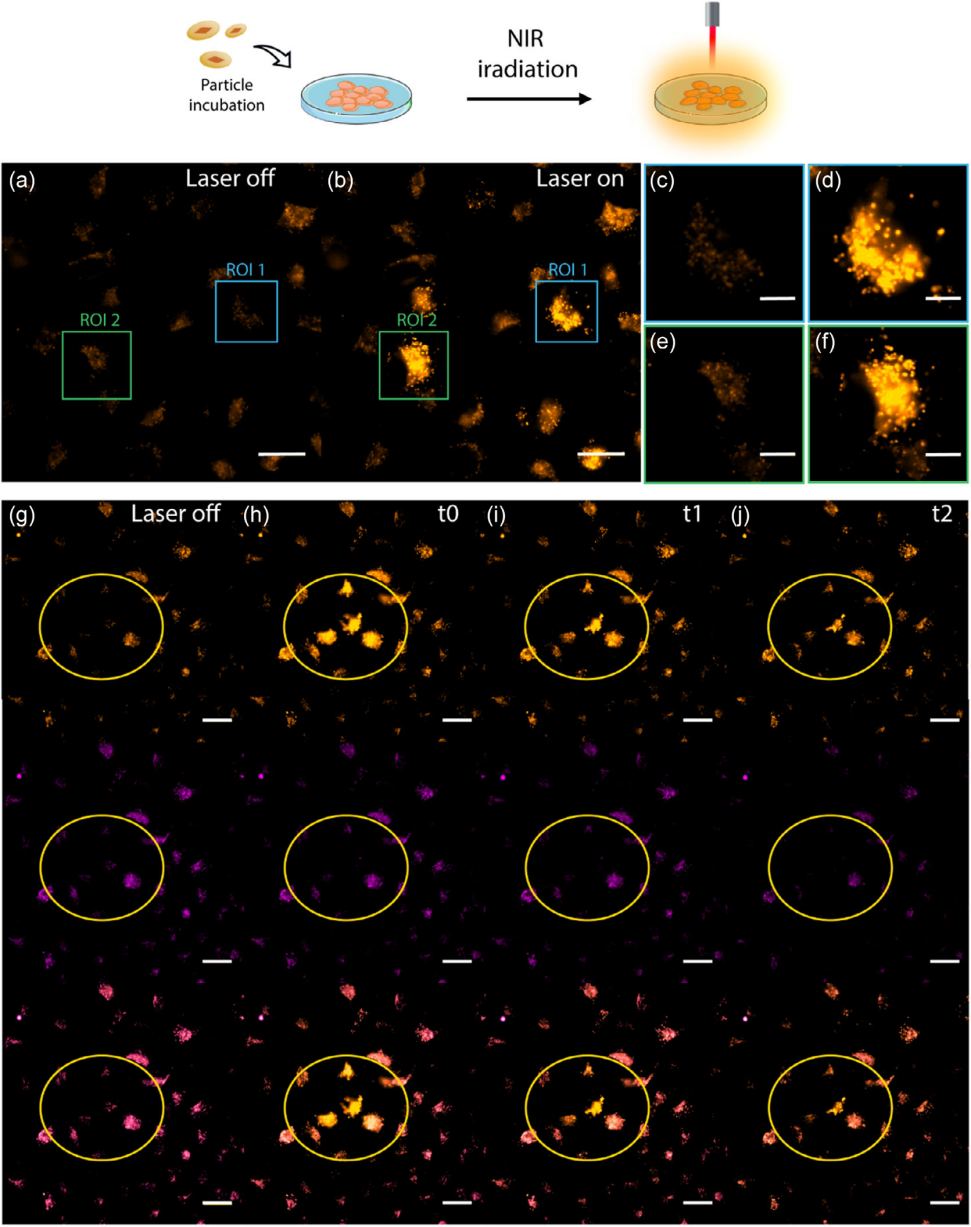
a) Microscopy images of cells incubates with AuBy@NU‐1000(CV)@PEG before NIR laser irradiation and b) after NIR laser irradiation in selected areas. Specific ROIs: c) ROI 1 and e) ROI 2 before irradiation; d) ROI 1 and f) ROI 2 after irradiation. g) Microscopy images show the fluorescence signal before NIR laser irradiation of the selected ROI, and sequential images at h) *t*0 = 10 s, i) *t*1 = 1 min, and j) *t*2 = 5 min after irradiation, highlighting the dynamic release of CV. The images feature the fluorescence signals from CV (orange), Lysotracker® (magenta), and the overlay of both channels. Scale bars are 25 μm. The samples were incubated with A549 cells for 6 h at a concentration of 100 pM.

As discussed previously and illustrated in Figure 4, CV remained enclosed within the pores of the NCs even after cellular uptake. After incubating cells with both types of NCs, individual cells were selected and exposed to NIR light to evaluate the induced release of encapsulated CV, as observed before and after irradiation (**Figure** **5**a,b and S21a,b, Supporting Information). To illustrate this process, magnified images of two regions of interest containing a single cell (ROI 1 and 2) are presented. These images show the cells before and after irradiation in Figure 5c,e and S21c,e, Supporting Information and in Figure 5d,f and S21d,f, Supporting Information, respectively. Following irradiation, these regions were immediately examined, revealing a pronounced increase in FI within the CV channel. This controlled release of CV is attributed to the local Δ*T* in the vicinity of the AuBy under NIR irradiation, leading to the release of CV encapsulated within the NC pores.

Furthermore, this behavior was analyzed over time by capturing multiple images at various time intervals after scanning the selected ROI (up to 5 min). These images revealed a noticeable diffusion of CV across the cell for both systems, with the signal diminishing after a few minutes compared to the immediate postirradiation signal (Figure 5g–j and S21g–j, Supporting Information). Over time, the FI of CV slightly decreases as the dye diffuses throughout the cell. Despite this gradual reduction, the FI remains noticeably higher in irradiated cells compared to nonirradiated ones, indicating a sustained release and presence of CV within the cell.

### 2.5 Drug Loading—Efficiency as DDSs

To further explore the potential of NCs as DDSs, NU‐1000‐based NCs were chosen due to their superior stability, porosity, and cell viability profile, in comparison to those based on ZIF‐8. The study involves the encapsulation of various chemotherapeutics (doxorubicin, carboplatin, and oxaliplatin, see Figure S22, Supporting Information) within the pores of the MOF to perform viability studies. For drug loading within the structure's pores, we employed a postsynthesis encapsulation method similar to the procedure used for the CV samples. Then particles were subsequently functionalized with PEG to prevent drug leakage.

Doxorubicin (DOX), an anthracycline, serves as a highly efficient antineoplastic agent extensively employed in the treatment of diverse cancers such as leukemia, ovarian cancer, and breast cancer.^[^ ^37^ ^]^ Carboplatin (CbPt), a platinum‐based chemotherapeutic agent, finds application in the treatment of ovarian, testicular, cervical, neck, head, and small cell lung cancers.^[^ ^38^, ^39^ ^]^ Oxaliplatin (OXA), a third‐generation platinum‐based drug, is commonly used as a first‐line chemotherapy for metastatic colorectal cancer.^[^ ^40^, ^41^ ^]^ Both carboplatin and oxaliplatin exert their therapeutic effects by binding to DNA, inhibiting transcription and replication processes, ultimately inducing cellular apoptosis.^[^ ^42^ ^]^ However, their clinical use is hampered by limitations such as low aqueous solubility and secondary effects.^[^ ^37^, ^42^ ^]^ Consequently, these chemotherapeutics would benefit from innovative systems capable of enhancing cellular uptake, drug accumulation at the tumor site, thereby augmenting therapeutic efficacy while mitigating associated toxicity.

**Figure 6:**
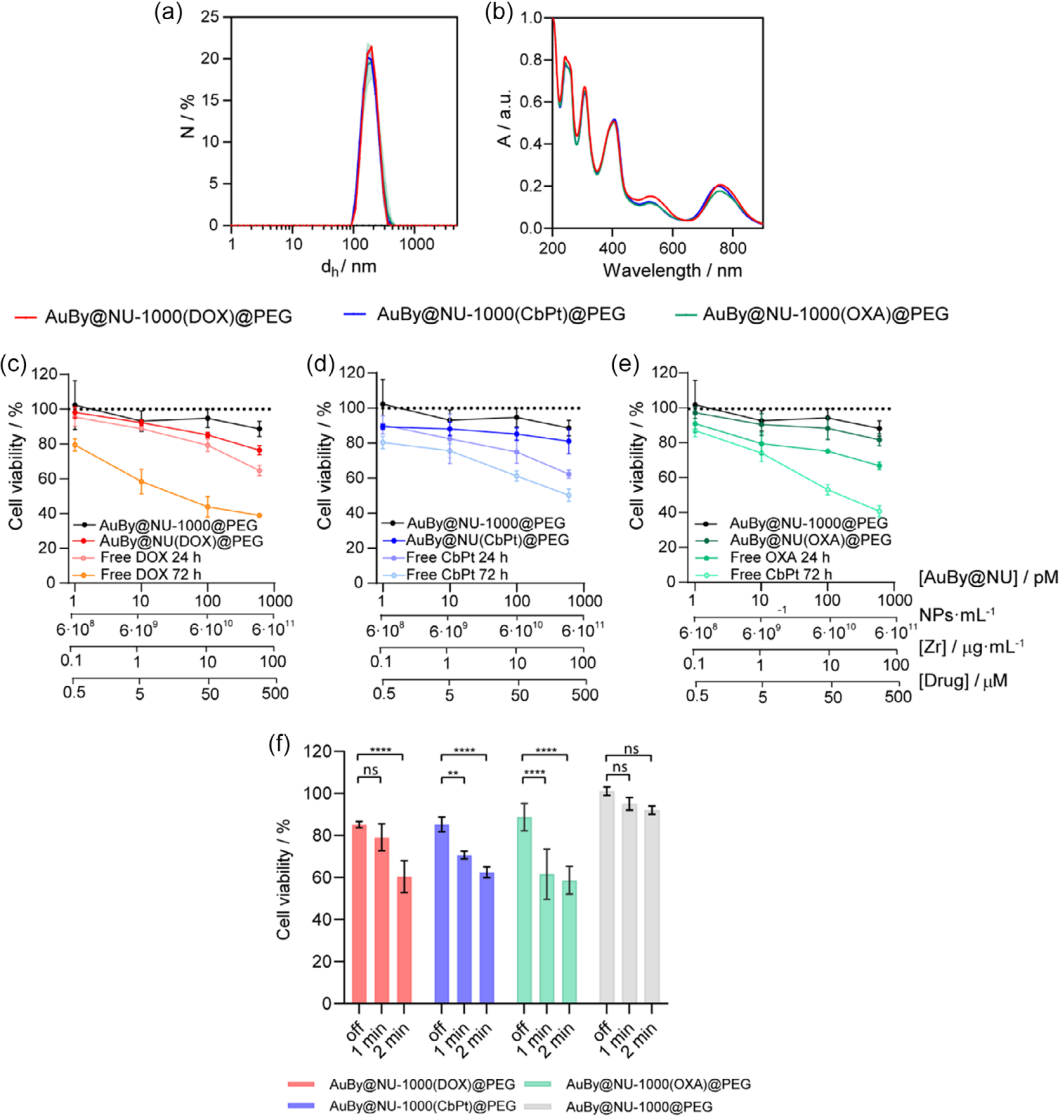
a) Number distribution of the hydrodynamic diameter *d*_h_ and b) UV–vis spectra of the drug‐loaded AuBy@NU‐1000 NCs. Cell viability using MTT assay of A549 cells 24 h after the incubation with the drug loaded NCs and the corresponding quantities of the free drug after 24 and 72 h of incubation c) DOX, d) CbPt, and e) OXA. f) Cell viability of A549 cells incubated for 6 h with the loaded NCs using MTT assay 24 h after the irradiation at a 20 W cm^−2^ irradiance (NC's concentration: 100 pM). Experiments were carried out using *n* = 4. Error bars represent standard deviation. Statistical analysis was assessed by two‐way ANOVA test (***p = 0.0018, ****p < 0.0001, ns = no significant difference).

The size of drug‐loaded NCs was characterized using DLS. As presented in **Figure** **6**a, the mean *d*_h_ does not present any significant differences among the samples (Table S6, Supporting Information). Similarly, UV–vis spectroscopy reveals no differences among samples, before and after the encapsulation/coating, leading to a final plasmon at 808 nm (Figure 6b). To further verify the size and determine the concentration of the NCs, we conducted NTA measurements (Figure S23, Supporting Information), which revealed no discernible differences among them. Additionally, we utilized inductively coupled plasma optical emission spectroscopy (ICP‐OES) analysis to evaluate the particle concentration, in combination with NTA values, and calculate the efficiency of drug loading for the Pt‐drugs (Table S7, Supporting Information). For DOX, encapsulation characterization was performed using fluorescence measurements. Fourier transform infrared (FTIR) spectra of the systems were analyzed (Figure S24, Supporting Information). The loading of each drug is clearly observed when compared with the spectra of the free molecules. DOX presents main peaks at 3310 and 2915 cm^−1^ corresponding to N—H and C—H stretch, respectively, that can be appreciated in the FTIR spectrum of the loaded NCs. On the other hand, for CbPt we can observe a main peak in 3270 cm^−1^ corresponding to N—H stretch that again can be subtly observed in the loaded MOFs. Finally, in OXA FTIR we could observe two peaks at 3515 and 3258 cm^−1^ related to O—H and N—H vibrations and a peak at around 1700 cm^−1^ related to the O—C═O section in the oxaliplatin structure, present also in the OXA encapsulated NCs. The PEGylation is confirmed with a vibration band at 2876 cm^−1^ in all samples.

Samples were further characterized using PXRD to confirm that crystallinity was maintained after drug encapsulation and PEGylation. The obtained diffraction patterns confirmed the presence of pure‐phase NU‐1000 in all cases (Figure S25, Supporting Information). These results are consistent with previous findings involving CV loading.

Then, the interaction between the NCs and cells was evaluated using 2D cell cultures of A549 cells. The biocompatibility was assessed through the MTT assay. NCs were incubated with A549 cells for 24 h at concentrations up to 600 pM (equivalent to a Zr concentration of 60 μg mL^−1^). In parallel, free drugs were incubated with the cells for both 24 and 72 h at concentrations of up to 500 μM, corresponding to the quantities encapsulated within the NCs (Figure 6c–f). A noticeable increase in the toxicity effect of the selected drugs becomes more evident following a 72 h incubation period.^[^ ^35^, ^39^, ^43^, ^44^, ^45^ ^]^ Upon comparing the toxicity of free versus encapsulated drugs after 24 h, cell viability was preserved when the drugs were encapsulated within the NCs. This underscores the effective surface functionalization with PEG, which mitigates cargo leakage. A slight increase in toxicity, particularly noticeable at the maximum dose of 600 pM compared to nondrug‐loaded NCs, is attributed to minor drug leakage. Altogether, these results prove that PEGylation of NCs retains the encapsulated drug more efficiently than observed in previous studies. For instance, Zhao and co‐workers reported toxicities exceeding 20% at concentrations of 300 μg mL^−1^ after 24 h incubation using non‐PEGylated DOX‐loaded NU‐1000 micrometric particles.^[^ ^35^ ^]^

The efficiency of these NCs as stimuli‐responsive DDSs was studied using a NIR irradiation setup, as described in previous work.^[^ ^19^ ^]^ NCs were incubated with A549 cells for 6 h at a concentration of 100 pM, equivalent to ≈50 μM of the drug, followed by irradiation at an irradiance of 20 W cm^−2^ for durations of 1 and 2 min. The irradiance was increased with respect to previous studies due to the differences observed between the initial release assessments (test tube), which utilized a 100 pM NMOF solution in an aqueous medium, and the subsequent in vitro irradiation studies. In these latter studies, despite the cells being treated with a 100 pM concentration, NMOF internalization is significantly lower (noninternalized NMOFs were removed). Consequently, to achieve a release effect comparable to the initial assessments, an increase in irradiance was necessary. Energy densities commonly used (i.e., 0.5 to 3 × 10^3^ J cm^−^^2^) are comparable to those employed in this work. Subsequently, MTT toxicity assays were conducted 24 h postirradiation to evaluate cell viability. Figure 4f presents results after 20 W cm^−2^ irradiance. The experiment was corroborated using a lower irradiation condition of 15 W cm^−2^ (Figure S26, Supporting Information). The results show a clear drop in cell viability under both irradiance conditions compared to nonirradiated samples (laser off), with higher cell death rates observed after 2 min of NIR exposure (Table S8–S10, Supporting Information). Nondrug‐loaded NCs demonstrated a nonsignificant reduction in cell survival rates postirradiation. Notably, the irradiation settings were precisely adjusted to initiate drug release while minimizing hyperthermia induced by the AuBy core. These settings are adequate to trigger the release of the encapsulated cargo without causing cell death associated with hyperthermia.

### 2.6 DDSs Efficiency Inside Living Cells in 3D Cell Culture

Spheroids and organoids are becoming essential for investigating the interactions between nanomaterials and living cells, increasingly supplanting the role traditionally occupied by 2D adherent cell cultures. These 3D cell models more accurately mimicking in vivo conditions compared to 2D models, becoming a reliable alternative to animal models in academic research and drug discovery.^[^ ^46^ ^]^ Spheroids, which are 3D multicellular aggregates of cells, exhibit properties that closely resemble those of solid tumors, including the formation of a necrotic center with limited access to oxygen and other metabolites.^[^ ^47^, ^48^ ^]^ These 3D cell cultures develop pH, oxygen, metabolic, and proliferative gradients, leading to stratification in mature spheroids. This stratification mirrors the vascular stages of solid tumors, effectively emulating the in vivo situation.

**Figure 7:**
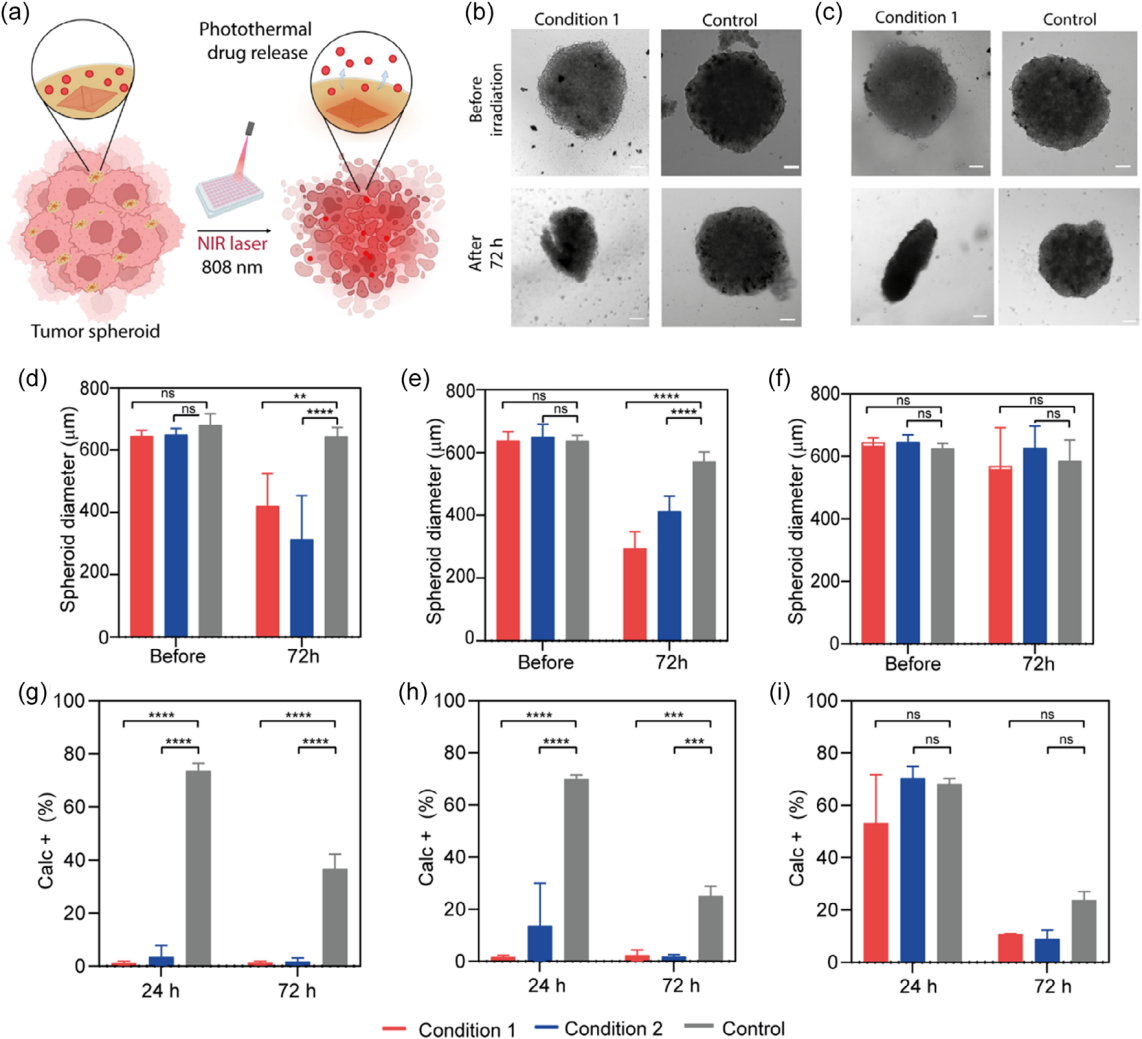
a) Scheme of work representing the degradation of the spheroids incubated with the NCs after the NIR irradiation. Microscopy images of the spheroids before and 72 h after the irradiation at condition 1 and control for b) AuBy@NU‐1000(CbPt)@PEG and c) AuBy@NU‐1000(OXA)@PEG. The spheroids diameter before and after 72 h of irradiation d) Aby@NU‐1000(CbPt)@PEG, e) AuBy@NU‐1000(OXA)@PEG, and f) AuBy@NU‐1000@PEG. Flow cytometry results of % of calcein stained cells after 24 and 72 h of irradiation for g) AuBy@NU‐1000(CbPt)@PEG, h) AuBy@NU‐1000(OXA)@PEG, and i) AuBy@NU‐1000@PEG. Experiments were carried out using *n* = 3. Error bars represent standard deviation. Statistical analysis was assessed by two‐way ANOVA test (***p = 0.006, ****p < 0.0001, ns = no significant difference).

In our study, we growth 3D spheroids from A549 cells as an in vitro model to closely replicate the in vivo tumor microenvironment. This model was employed to evaluate the interactions with our NIR‐responsive AuBy@NU‐1000@PEG NC, loaded with chemotherapeutics (**Figure** **7**a), analogously to our previous investigations in 2D cultures. Three days after seeding the cells, confocal microscopy was used to examine the morphology of the A549 spheroids. Under the experimental conditions employed, spheroids of ≈600 μm in diameter were obtained (Figure 7b,c). Building on our previous findings and optimized protocols for studying the interaction of NCs and spheroids,^[^ ^26^ ^]^ CbPt‐ and OXA‐loaded NU‐1000‐based NCs were incubated with A549 spheroids at a NC's concentration of 100 pM (equivalent to a drug concentration of 50 μM) for 24 h. After incubation, the samples were washed thoroughly and then irradiated with the NIR setup previously described. The conditions for the NIR irradiation of 3D cell models were optimized. 20 W cm^−2^ in two 1 min pulses (Condition 1, Table S11, Supporting Information) was initially applied to suit the robustness and depth of the spheroids.^[^ ^10^, ^49^ ^]^ Additionally, an alternative condition of 30 W cm^−2^ in two 30 s pulses was applied, yielding equivalent results, as shown in Figure S26–S30, Supporting Information (Condition 2, Table S11, Supporting Information). 24 and 72 h after NIR irradiation, spheroids were analyzed by microscopy and flow cytometry. The selected irradiances were optimized to achieve the intended outcome (drug release, as opposed to photothermal therapy).

First, fluorescence microscopy studies were performed both before and 72 h after irradiation (Figure 7b,c and S27–S31, Supporting Information) to evaluate the size of the spheroids after different treatments.^[^ ^26^ ^]^ Spheroids treated with AuBy@NU‐1000@PEG NCs loaded with CbPt (Figure 7b and S27, Supporting Information) and OXA (Figure 7c and S28, Supporting Information) show a decrease in size 72 h after NIR irradiation. Additionally, the experiment included free CbPt and OXA at concentrations (≈50 μM) equivalent to those encapsulated within the NCs, alongside control spheroids for comparison are shown in Figure S29–S31, Supporting Information. Figure 7d–f and S32, Supporting Information summarize the mean diameter of the spheroids in each scenario, as determined from the microscopy images. The microscopy images, supported by the measured mean diameters, unequivocally show a significant reduction in the size of the spheroids 72 h postirradiation in samples incubated with drug‐loaded NCs. In contrast, no changes in morphology or size were observed in spheroids treated with nonloaded NCs after NIR irradiation, indicating no significant phototoxicity effects of the applied irradiance (Figure S29, Supporting Information). This indicates that the thermoplasmonic effect alone is insufficient to achieve cell death under the selected irradiation conditions, as previously demonstrated in 2D cell cultures (Figure 6f). Notably, the obvious size reduction observed with drug‐loaded NCs can be directly attributed to the controlled drug release, enabled by photothermal activation. In general, precedents in the literature typically combine hyperthermia and photothermal‐driven drug release to achieve cell death.^[^ ^50^, ^51^ ^]^

Second, to corroborate the effect of CbPt and OXA release in 3D spheroids treated with drug‐loaded NCs after NIR irradiation, a live‐dead assay was carried out by flow cytometry using calcein‐AM and propidium iodide (PI) as fluorescence indicators for viable and dead cells, respectively. Cytometry studies of treated spheroids were conducted 24 and 72 h postirradiation (Figure 7g–i and S33–S35, Supporting Information). Viable cells exhibit strong green fluorescence, resulting from the conversion of calcein‐AM into calcein by cellular esterases. Conversely, PI is a nuclear staining dye that cannot penetrate the membranes of viable cells and thus stains dead (necrotic) cells with red fluorescence. The total percentage of calcein‐positive cells (viable cells) is presented in Figure 7g–i and S35, Supporting Information. Elevated percentages of PI staining were observed in all cases, a phenomenon attributable to the necrotic core of the spheroids.^[^ ^52^, ^53^ ^]^

Decrease viability assessed by calcein staining (less than 2% of viable cells) was shown in spheroids treated with drug‐loaded NCs after 24 h postincubation (Figure 7g,h), indicating that nearly all the remaining cells were dead or beginning to die. In contrast, spheroids incubated with nondrug‐loaded NCs, regardless of whether they were irradiated or not, show an equivalent percentage of viable cells after 24 h (Figure 7i), which is also comparable to that of nonirradiated, drug‐loaded NCs. These results suggest a negligible effect attributable to photothermal hyperthermia, which has been previously employed to cause cell death in 3D cell models.^[^ ^54^, ^55^ ^]^ The difference in outcomes between spheroids treated with free drugs and control spheroids, irrespective of whether they were irradiated or not, is negligible (Figure S35, Supporting Information). This aligns with previous findings, indicating that the quantities of CbPt and OXA employed are not expected to induce toxicity within 24 h.^[^ ^43^, ^45^ ^]^

These findings indicate that 24 h posttreatment, the drug‐loaded NCs, when coupled with NIR irradiation, result in significant treatment efficacy achieving high sensitivity to CbPt and OXA. This efficacy is attributed to the rapid NIR‐controlled diffusion of the drug throughout the spheroid. In contrast, the permeability and efficiency of the drugs are lower without the NCs. Similar effects were observed after 72 h for the drug‐loaded NCs, with a noticeable reduction in cell viability in the controls, as depicted in Figure 7g–i and S35, Supporting Information. It is noted that the hyperthermia effect induced by the irradiated empty NCs can lead to cell death after 72 h, and free drugs also begin to exhibit some level of toxicity after this time frame, regardless of irradiation.

Altogether, these findings suggest that the proposed NIR‐responsive NCs could serve as nanocarriers, facilitating the rapid internalization of loaded drugs and enabling their effective, triggered release.

## 3 Conclusion

In this study, we explored two core@shell NCs featuring a AuBy nanocore embedded in NMOFs, either ZIF‐8 or NU‐1000. These NCs not only preserved the plasmonic properties inherent to AuBy but also exhibited exceptional cargo‐loading capabilities within their microporous MOF nanoshells. To improve stability and mitigate unspecific cargo leakage, both systems were enhanced with polymer functionalization. Subjected to NIR irradiation, the NCs demonstrated remarkable photothermal properties, enabling controlled cargo release through thermoplasmonic activation. In vitro testing confirmed the efficacy of these NIR‐responsive NCs as efficient DDSs, with drug‐loaded NU‐1000/NCs showing promising results in both 2D and 3D cell cultures.

In conclusion, our study signifies substantial progress in the creation of smart, stable, and biocompatible core@shell NMOF platforms. These platforms excel as robust vehicles for intracellular drug delivery, merging thermoplasmonic capabilities with outstanding cargo‐loading capacities. Our results position these NCs at the forefront of advanced nanomaterials for bioapplications, establishing them as potential tools in advancing drug delivery techniques within nanomedicine.

## 4 Experimental Section

4.1

4.1.1

AuBy synthesis,^[^ ^25^ ^]^ PEGylation,^[^ ^56^ ^]^ phase transfer of AuBy,^[^ ^57^ ^]^ synthesis of NU‐1000,^[^ ^21^ ^]^ PEGylation of AuBy@NU‐1000,^[^ ^30^ ^]^ and AuBy@ZIF‐8^[^ ^16^ ^]^ were adapted from previous reports. Milli‐Q water, with a resistivity of 18 MΩ cm at 25 °C, served as the solvent in all conducted experiments and for the rinsing of glassware. All reagents were utilized without additional purification.

##### Materials

Silver nitrate (AgNO_3_, ≥99.0%); phosphorus oxychloride (POCl_3_, 99%); poly(ethylene glycol) methyl ether (MeO‐PEG, 5 kDa); poly(ethylene glycol) methyl ether thiol (MeO‐PEG‐SH, 2 kDa); triethylamine (≥99.5); l‐ascorbic acid (C_6_H_8_O_6_, ≥99%); sodium borohydride (NaBH_4_, ≥98.0%); citric acid (≥99.5%); cetyltrimethylammonium chloride (CTAC, 25 wt% in H_2_O); CTAB (≥98%); poly(isobutylene‐alt‐maleic anhydride) (average *M*_w_ ≈6,000, 12–200 mesh 85%); doxorubicin hydrochloride (C_27_H_29_NO_11_ HCl, 98.0–102.0% HPLC); zinc nitrate hexahydrate (Zn(NO_3_)_2_•6H_2_O ≥ 98%); zirconyl chloride octahydrate (ZrOCl_2_ 8H_2_O, 98%), and 2‐methylimidazole (MeIm, 99%) were provided by Sigma–Aldrich. Hydrogen tetrachloroaurate (III) hydrate (HAuCl_4_, Au≥49%); dichloromethane (DCM, ≥99.8%); hydrochloric acid (HCl, 37%); DMF (≥99.8%) and chloroform (CHCl_3_, ≥99.8%); acetic acid glacial (CH_3_COOH, 99.7%); DMF (≥99.8%) were provided by Thermo Scientific.

Oxaliplatin (C_8_H_14_N2O_4_Pt) and carboplatin (C_6_H_12_N_2_O_4_Pt, >98%) were provided by TCI Chemicals. CV acetate (C_18_H_15_N_3_O_3_) was provided by Alfa Aesar. 1,3,6,8‐Tetra(4′‐carboxyphenyl)pyrene) (H_4_TBAPy) (C_44_H_26_O_8_, 95%) was provided by Alfachem.

##### Generation of Pentatwinned Seeds

Within a 20 mL glass vial, initial seeds were prepared by combining 8.639 mL of water with 0.661 mL of the CTAC solution and 0.100 mL of 25 mM HAuCl_4_ (in water) under vigorous magnetic stirring (1000 rpm) for 5 min. Subsequently, 0.1 mL of 0.5 M citric acid (in water) was introduced into the reaction mixture, followed immediately by two consecutive doses of 0.25 mL of freshly prepared NaBH_4_ 25 mM (in water). The solution presented a color change from pale yellow to a brownish solution, indicating the formation of gold seeds. After 2 min of stirring, the vial was sealed, and the seeds were subjected to heating in a block at 80 °C for 90 min under gentle stirring (300 rpm). Ultimately, the solution's color transitioned from brown to red, signifying the successful creation of pentatwinned defects through the aging process.

##### AuBy Synthesis

Within a 600 mL glass bottle, the growth solution for AuBy was formulated by introducing, under magnetic stirring (500 rpm), 275 mL of water, 250 mL of 0.2 M CTAB, 10 mL of 25 mM HAuCl_4_, 5 mL of 10 mM AgNO_3_, 0.833 mL of HCl, and 4 mL of 0.1 M ascorbic acid (all in water). Following the color transition from orange‐yellow to colorless upon the addition of ascorbic acid, 12 mL of pentatwinned seeds was incorporated. The reaction mixture was maintained at 30 °C for 2 h. Subsequently, the NPs were washed three times with water to eliminate excess CTAB and were then retained in a final volume of 10 mL of water.

##### PEGylation and Phase Transfer of AuBy

In the absence of excess CTAB, AuBy exhibits a LSPR at ≈808 nm, with an absorption molar coefficient of 3.63 × 10^10^ M^−1^ cm^−1^ at this wavelength. For PEGylation, 500 000 MeO‐PEG‐SH (2 kDa) chains were utilized per particle. A 1% fraction of this total (5000 chains) was introduced into the colloidal dispersion of AuBy, equating to the addition of 75 μL of 20 mg mL^−1^ MeO‐PEG‐SH solution to 10 mL of 15 nM AuBy in water. In a separate tube, 150 mg of MeO‐PEG‐SH, corresponding to 500 000 chains per particle, was dissolved in chloroform. The NPs in water and the MeO‐PEG‐SH solution in chloroform were combined in a separation funnel, vigorously shaken, and followed by the addition of 10 mL of methanol to initiate phase transfer from the aqueous to the organic phase. The resulting emulsion was disrupted with the addition of more water. Once AuBy were transferred to the organic phase, they were collected, dried under rotary evaporation, and subsequently redispersed in the same volume of DMF as the initial volume.

##### Synthesis of AuBy@NU‐1000 NCs

In a 20 mL glass vial, a mixture was prepared by dissolving 64 mg of ZrOCl_2_·8H_2_O and 10 mg of H_4_TBAPy in 9 mL of DMF. Subsequently, 1 mL of PEGylated AuBy at a concentration of 10 nM was introduced into the solution, followed by the addition of 2 mL of acetic acid and 1 mL of water. The vial was sealed and subjected to heating at 90 °C for 30 min. Following this, the resulting product was centrifuged at 7200 rcf for 10 min, with three subsequent washes using DMF and three washes with acetone. The product was then dried under vacuum for subsequent characterizations. NU‐1000 formula: C_88_H_48_O_32_Zr_6_.

##### Synthesis of AuBy@ZIF‐8 NCs

The CTAB‐stabilized AuBy served as seeds for the growth of a ZIF‐8 shell. In a 5 mL glass vial, under magnetic stirring at room temperature, a mixture was created by combining 1 mL of a 1.3 M aqueous solution of 2‐methylimidazole, 1 mL of a 0.025 M aqueous solution of zinc nitrate hexahydrate, and 1 mL of a 1.5 nM AuBy dispersed in a 0.16 mg mL^−1^ CTAB aqueous solution. The mixture was stirred for 5 min and left to stand for 3 h at room temperature. The development of purple turbidity signified the formation of AuBy@ZIF‐8. Subsequently, the particles were washed by centrifugation at 7200 rcf for 10 min and were rinsed twice with methanol. ZIF‐8 formula: C_8_H_10_N_4_Zn.

##### CV Loading

Dispersions of AuBy@NU‐1000 and AuBy@ZIF‐8 NCs, comprising 200 μL with a solid concentration of 1 mg mL^−1^ in MeOH, were combined with a 200 μL solution of CV in water at a concentration of 2 mg mL^−1^. The resultant mixture was kept overnight incubation at room temperature with continuous shaking. Postincubation, NCs were separated from the solution through centrifugation at 7200 rcf for 10 min.

##### PMA Synthesis

An amphiphilic polymer, poly(isobutylene‐alt‐maleic anhydride)‐graft‐dodecyl, was synthesized following a protocol previously described by Parak et al.^[^ ^56^ ^]^ For clarity, after modification with fluorescein amine (FAM) molecules, this polymer is referred to as PMA, endowing it with fluorescence properties. In a 50 mL round flask, 20 mmol of poly(isobutylene‐alt‐maleic anhydride), 15 mmol of dodecylamine (DDA) and 0.4 mmol of FAM‐amine were mixed in 40 mL of tetrahydrofuran (THF). The solution was kept under magnetic stirring (350 rpm) and reflux (65 °C) overnight. Then, the THF was evaporated under vacuum using a rotary evaporator and the dried polymer was redispersed in 10 mL of chloroform. To purify the polymer and remove the excess of unreacted DDA, 50 mL of hexane was added to the solution and left for 2 h in the freezer (−4 °C). The sample was centrifuged (2 min, 7100 rcf) to remove the hexane and dried under vacuum. The powder was weighed in order to prepare a final solution in chloroform of a monomer (MW = 154 g mol^−1^) concentration 0.5 M.

##### Synthesis of mPEG‐PO_3_

mPEG‐PO_3_ was synthesized through a modified adaptation of a previously documented procedure.^[^ ^58^ ^]^ The synthesis transpired in a two‐neck flask, initiating with the addition of 50 mmol of POCl_3_ dissolved in 5 mL of DCM. Subsequently, 150 mmol of triethylamine was introduced at 0 °C. After 30 min, 15 mmol of mPEG (5 kDa) was incorporated into the mixture in 50 mL of DCM. The resulting solution was stirred at room temperature for 10 h. After the stirring phase, 20 mL of water was introduced to the reaction mixture and allowed to react for an additional 3 h. Following this, the DCM solvent was removed under vacuum using a rotavapor, and the crude product was purified through dialysis, employing a dialysis membrane with a molecular weight cutoff of 3500 Da. Ultimately, the remaining water content was eliminated through lyophilization, resulting in the production of mPEG‐PO_3_ in the form of a white solid.

##### AuBy@ZIF‐8(CV) PMA Coating

NCs as dispersed in MeOH were mixed with PMA in chloroform solution (600 monomers per nm^2^ surface area of NP, considering for the calculations a sphere with the same NC diameter, and the solvent (3:1 MeOH:CHCl_3_) was slowly evaporated in a rotary evaporator. Then the dried product was resuspended by adding ≈0.5 mL of sodium hydroxide (0.1 M, pH 13), aided by sonication (1 min). The resulting NPs (AuBy@ZIF‐8(CV)@PMA) were collected by centrifugation (7000 RCF, 10 min), washed twice with sodium hydroxide (0.01 M, pH 12), and finally redispersed in water.

##### AuBy@NU‐1000(CV) PEGylation

Surface modification of AuBy@NU‐1000(CV) employed the mPEG‐PO_3_ polymer. For this, the product from a synthesis previously loaded with CV AuBy@NU‐1000(CV) in water was combined with 2.5 mL of a 10 mg mL^−1^ aqueous solution of mPEG‐PO_3_. The resulting mixture was stirred using a magnetic bar at 500 rpm for 3 h. Subsequently, the coated material was washed 3 times with water by centrifugation at 10 000 rcf for 10 min.

##### Quantification by Fluorescence Measurements

Fluorescence characterization in solution was performed using a Horiba FluoroMax‐3 spectrometer. The amount of CV molecules loaded into NCs was quantified by fluorescence (*λ*_exc_/*λ*_em_ = 590/620 nm) indirectly, by measuring the CV remaining in the SN after centrifugation and washing steps of the nanosystems. The CV concentration in the SN was determined by interpolation of the measured FI to previously constructed analytical calibration curves.

##### SEM

SEM micrographs were obtained using a ZEISS FE‐SEM ULTRA Plus after the deposition of a drop of diluted sample onto a piece of clean silicon wafer.

##### Thermogravimetric Analysis

*Thermogravimetric analysis* measurements were carried out using a TA Instruments Inc. SDT Q‐600 thermobalance with a general heating profile from 25 to 800 °C and a heating rate of 5 °C min^−1^ under air using a flux of 100 mL min^−1^. Before the analysis, all the samples were lyophilized and dried at 100 °C.

##### UV–vis/Fluorescence Spectroscopy

UV–vis absorption spectra were recorded using an Agilent UV–vis Cary 3500 spectrophotometer. The measurements were performed using a 1 cm quartz cell at a controlled temperature of 25 °C to minimize variability. Data acquisition was carried out over a wavelength range of 200–1000 nm to capture the entire extinction profile.

##### NTA

A NanoSight NS300 (Malvern Instruments, UK) equipped with a 488 nm laser module, a sCMOS camera, and a syringe pump was used for all NTA measurements. All measurements were carried out at 24 °C. NCs were diluted in filtered MilliQ water to a final volume of 1 mL and loaded in the measurement chamber with a flow rate of 100 μL min^−1^. Flow mode measurements were obtained recording three videos of 60 s for each measurement. The NanoSight NS300 software was used to analyze the sample (10–100 particles/frame). Concentration of NPs in pM was calculated using NTA measurements, where the obtained results in particles·mL^−1^ were converted to M by the relation:

(1) $$\frac{\text{mol NPs }}{L}=\frac{\text{Particles}}{\text{mL}}\text{ × }\frac{1\text{ mol NPs}}{N_{A}\text{ particles}}\text{ × }\frac{1000\text{ mL}}{1\text{ L}}$$

##### Dynamic Light Scattering

The hydrodynamic diameter (*d*_h_) and polydispersity index of the NPs were determined via DLS using a Malvern Zetasizer Nano ZSP equipped with a 10 mW He–Ne laser operating at a wavelength of 633 nm and a fixed scattering angle of 173°.

##### N_2_ Adsorption–Desorption Analysis

N_2_ sorption isotherms at 77 K were measured on a Micromeritics 3Flex Adsorption Analyzer. The samples (about 20–30 mg) were activated overnight under a high vacuum at 90 °C prior to analysis. The specific surface area was extrapolated within the relative pressure (*P*/*P*_0_, where *P*_0_ is the saturation pressure) interval of 0.05–0.3 by applying the BET equation. The data were analyzed using the 3Flex V5.03 software (Micromeritics Instrument Corp., Norcross, GA, USA).

##### Colloidal Stability Tests

DLS measurements at different times were carried out in different media: MilliQ water and complete cell culture medium (DMEM with phenol red, 4.5 g l‐1d‐glucose, l‐glutamine and pyruvate (DMEM Gibco, Thermo Fisher Scientific, Massachusetts, USA) supplemented with 10% fetal bovine serum (Gibco, Thermo Fisher, Massachusetts, USA) and 1% penicillin/streptomycin (P/S, Gibco, Thermo Fisher Scientific, Massachusetts, USA)).

##### ICP‐OES

The elemental analysis was performed using an Agilent 5800 ICP‐OES. Prior to analysis, the samples underwent acid digestion through Anton Paar Multiwave GO Plus microwave heating system. Calibration curves ranging from 0 to 10 ppm were constructed for all the analyzed elements (Zr, Zn, Pt, and Au). Additionally, Mn and Se were added at known concentrations as internal standards. For each element, the concentration was extrapolated from two atomic emission lines: Zr at 339.198 and 343.823 nm, Zn at 202.548 and 213.857 nm, Pt at 203.646 and 214.424 nm, Au at 242.794 and 267.594 nm, Mn at 257.610 and 259.372 nm, and Se at 196.026 and 203.985 nm.

##### Cell Culture

The A549 (human lung carcinoma) cell line was maintained in culture in DMEM with phenol red, 4.5 g l‐1D‐glucose, l‐glutamine and pyruvate (DMEM Gibco, Thermo Fisher Scientific, Massachusetts, USA) supplemented with 10% fetal bovine serum (Gibco, Thermo Fisher, Massachusetts, USA) and 1% penicillin/streptomycin (P/S, Gibco, Thermo Fisher Scientific, Massachusetts, USA) (cDMEM). Cells were cultured at 37 °C under a 5% CO_2_ atmosphere and kept under humid conditions. After reaching 80% confluency, the cells were washed with Dulbecco's phosphate buffered saline (PBS, Thermo Fisher #14190169) and passaged after incubation with 0.25% Trypsin–EDTA (Gibco, Thermo Fisher Scientific, Massachusetts, USA).

##### MTT Assay

MTT (3‐[4,5‐dimethylthiazol‐2‐yl]‐2,5‐diphenyltetrazolium bromide) assay was performed to study cells viability after NCs exposure. A549 cells were seeded in 96‐well plates (7500 cells/well in 100 μL of cDMEM). After 24 h, the medium was removed and 100 μL of NCs solution diluted in fresh cDMEM were added. After 1 h, each well was rinsed once with 1X PBS and 100 μL of freshly prepared MTT solution diluted in cDMEM was added. 96‐well plates were left 3 h at 37 °C and 5% CO_2_, then MTT solution was removed, and 50 μL of DMSO were added to each well to dissolve the formazan crystals. After 10 min at 37 °C and 5% CO_2_, absorbance was measured with a plate reader (TECAN, Infinite® 200 PRO) at 540 nm. The absorbance value of each well provided by the instrument is an average of nine consecutive measures in the same well. Final absorbance value for control cells (*A*_c_) (untreated), is an average of, at least, nine different well values. Final absorbance values for samples (*A*_s_) are a mean of three independent well values. Cell viability value is calculated as follows: (*A*_s_/*A*_c_) × 100.

##### Laser Irradiation (Heating Experiments)

Experiments were conducted in 96‐well plates, depending on the experiment each well will contain NCs, cells, and NC‐exposed cells. Irradiation was performed using an 808 nm laser (Lasing, #FC‐W‐808A) coupled to a zoom fiber collimator (Thorlabs, #ZC618SMA‐B). This collimator was utilized to control the spotsize and ensure homogeneous irradiation of the cells. To determine the intensity of the laser irradiation received by the cells, a power energy meter (Thorlabs, #PM100D) equipped with a thermal powerhead (10 W, 25 mm, Thorlabs, #S425C) was employed to measure the output power. The spot size was determined by using a viewing card (Thorlabs, #VRC4) to visualize the spot, and ImageJ software was utilized to measure its dimension. As the beam was collimated, a uniform spot distribution was assumed, allowing the intensity to be calculated by dividing the power (7.85 W) by the surface area (0.785 cm^2^) of the spot giving an irradiance of 10 W cm^−2^. Various experimental conditions involving different time intervals and power densities were investigated, as specified in each individual experiment.

##### Microscopy Imaging

A549 cells were initially seeded onto μ‐slide 8 well‐IbiTreat chambers, with each well providing a surface area of 1 cm^2^. The seeding density employed was 20 000 cells per well, cultured in cDMEM for 24 h. After the 24 h incubation period, the cDMEM was replaced with freshly prepared solutions of ZIF‐8‐based NCs or NU‐1000‐based NCs at a concentration of 50 and 100 pM, respectively, diluted in cDMEM. Following a 3 h exposure to these particles, the cells underwent a single rinse with 1X PBS. This step aimed to eliminate any noninternalized particles that might have remained dispersed in the cDMEM prior to the subsequent image acquisition. Fluorescence images of the cells were captured using a Leica Microscope type DMI8, equipped with a Leica DFC9000 camera, and maintained in an incubator at a temperature of 37 °C under a 5% CO_2_ atmosphere. Cells were observed in the brightfield channel, the CV channel (wavelengths: excitation range 542–566 nm; emission range 578–610 nm), fluorescein channel (excitation range 462–496 nm; emission range 506–632 nm), NU‐1000 fluorescence channel (excitation range 375–407 nm; emission range 420–450 nm), and LysoTraker Deep‐red channel (excitation range 622–654 nm; emission range 666–724 nm).

To analyze the images, ImageJ software was utilized for image processing and data extraction. NIR irradiation of the samples was performed using Leica infinity scanner installed in the microscope that allows the scanning of the selected ROIs with a NIR laser using LASX software.

##### Spheroids

A549 cell lines were used to obtain the 3D spheroids. 100 μl of suspension of 50 000 cells·mL^−1^ was transferred to a 96 multiwell plate previously treated with agarose as follows: a 1–1.5% agarose solution was prepared in filtered PBS 1X and heated up to 100 °C; 50 μL of agarose solution were used to cover the bottom of the wells. The plate was let to cool at RT in sterile conditions without the lid for at least 30 min before seeding the cells. The 3D spheroids were grown at 37 °C in a humidified 5% CO_2_ atmosphere, with 100 μL of fresh complete media gently added every day, for 3 days in order to obtain spheroids with an ≈0.5 μm diameter. On the fourth day, the spheroids were washed with PBS 1X twice, carefully avoiding displacing them from the wells. The spheroids were incubated with a 200 μL solution of the NCs. After 24 h, the NCs solution was removed, and the spheroids were washed twice with PBS 1X. After that, the consecutive irradiation experiments were performed.

##### Live/Dead Assay

Each spheroid was individually transferred into the 96 multiwell plate without agarose. Then, they were harvested after trypsinization for 2 min with 0.05 mL of 0.25% Trypsin–EDTA. 0.2 mL of cDMEM was added to recover the cells, which were transferred to 96 MW V‐bottom plate. The cells were collected after centrifugation at 500 g for 5 min. The cell pellets were resuspended in 0.2 mL of PBS 1X at 0.1 μM calcein AM and 3 μM PI. The cells were incubated for 15–20 min at room temperature in the dark. After the incubation period, the stained cells were analyzed by flow cytometry (Beckman Coulter Cytoflex S B‐R‐V‐Y Series, C02947) using a 488 nm excitation and measuring green fluorescence emission of calcein (i.e., 530/30 bandpass) and red fluorescence emission of the PI (i.e., 610/20 bandpass). Results were analyzed using BD FlowJo 10.10.0.

##### Statistical Analysis

All data are presented as the mean and standard deviation. Experiments were carried out using *n* = 3. Error bars represent standard deviation. Statistical analysis was assessed by two‐way ANOVA test. Statistical significance was set at *p* < 0.05. All statistical analysis were conducted using GraphPad Prism 8.0.1.

## Conflict of Interest

The authors declare no conflict of interest.

## Author Contributions

M.C.‐M. and M.C. carried out the synthesis and characterization of the materials. M.C.‐M. and E.S. performed cell studies. G.Z. performed experimental work involving ICP‐OES. The manuscript was written through the contributions of all authors. All authors have approved the final version of the manuscript.

## Supporting information

Supplementary Material

## Data Availability

The data that support the findings of this study are available from the corresponding author upon reasonable request.
