# Supplementary material for: Light‐Responsive Nanoantennas Integrated into Nanoscale Metal–Organic Frameworks for Photothermal Drug Delivery
Source: Small Sci. 2024 May 10;4(8):2400088. doi: 10.1002/smsc.202400088 (PMC11935178; doi:10.1002/smsc.202400088)
Supplement: Supplementary file 1 — Supplementary Material [file SMSC-4-2400088-s001.pdf]

Supporting Information

Light-Responsive Nanoantennas Integrated into Nanoscale Metal-Organic Frameworks for Photothermal Drug Delivery

Manuela Cedrún-Morales, Manuel Ceballos, Enrica Soprano, Giulia Zampini, Ester Polo, Beatriz Pelaz and Pablo del Pino**

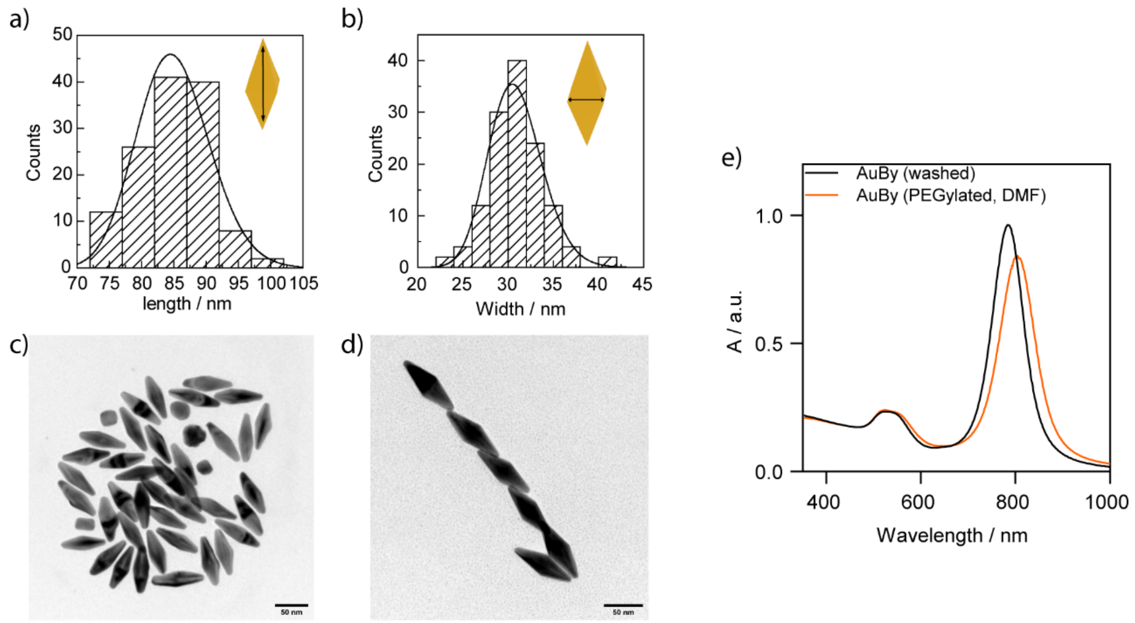

Figure S1: SEM analysis of AuBy. Size histograms corresponding to the a) length and b) width of the AuBy. SEM images of the c) Washed AuBys (used for AuBy@ZIF-8 synthesis) and d) PEGylated AuBys (used for AuBy@NU-1000 synthesis). e) UV-Vis spectra of the washed AuBys in water and after PEGylation and phase transfer to DMF. Scale bars correspond to 100 nm.

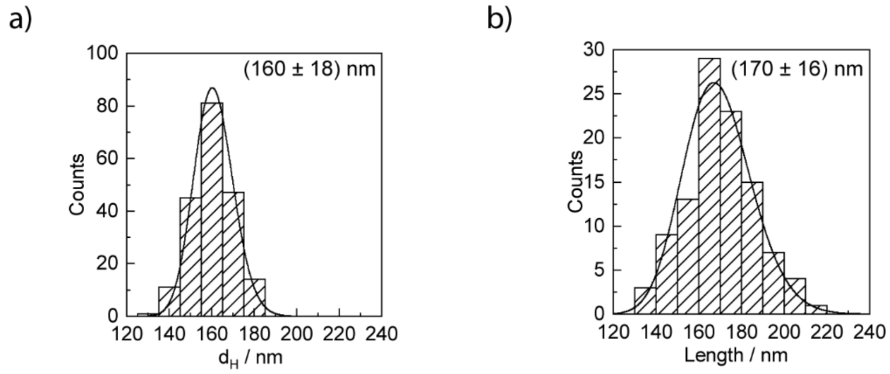

Figure S2: SEM analysis of both NCs. Size histograms corresponding to the diameter of a) AuBy@ZIF-8 and b) length of AuBy@NU-1000.

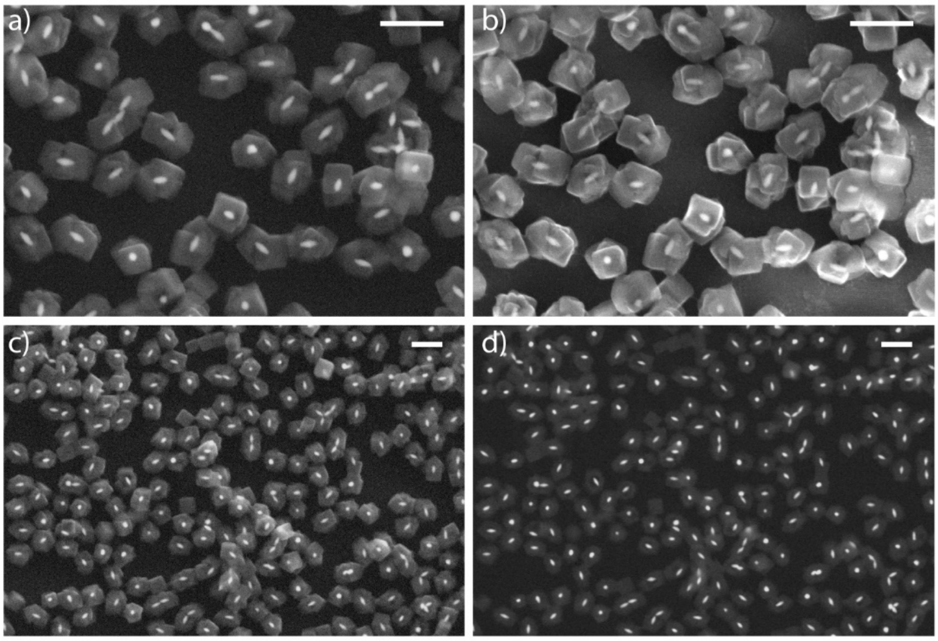

Figure S3: SEM images of a, b) AuBy@ZIF-8(CV) and c, d) AuBy@ZIF-8(CV)@PMA NCs. Scale bars correspond to 200 nm.

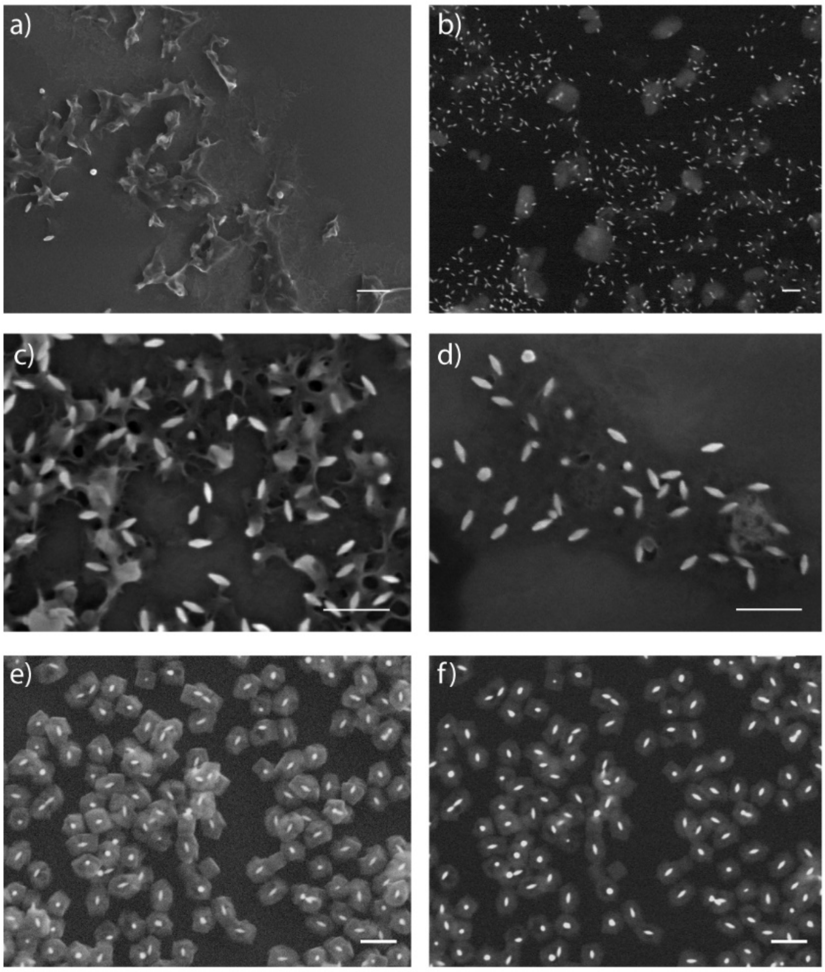

Figure S4: SEM images of the AuBy@ZIF-8 NCs placed in water before the PMA coating for a-b) 5h, c-d) 24h, and after the PMA coating for e-f) 7 days.

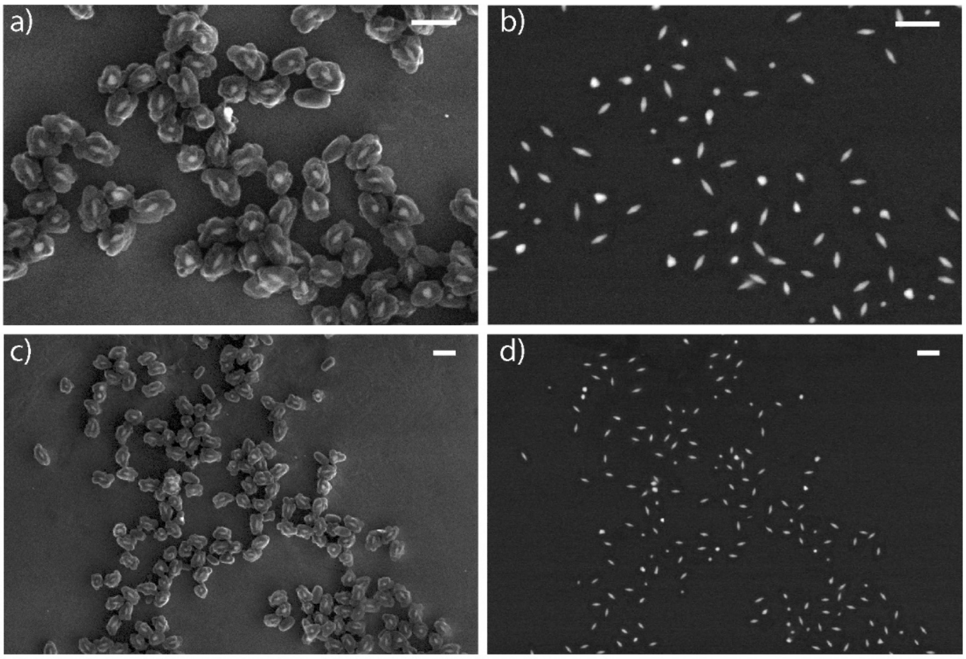

Figure S5: SEM images of a,b) AuBy@NU-1000(CV) and c,d) AuBy@NU-1000(CV)@PEG NCs. Scale bars correspond to 200 nm.

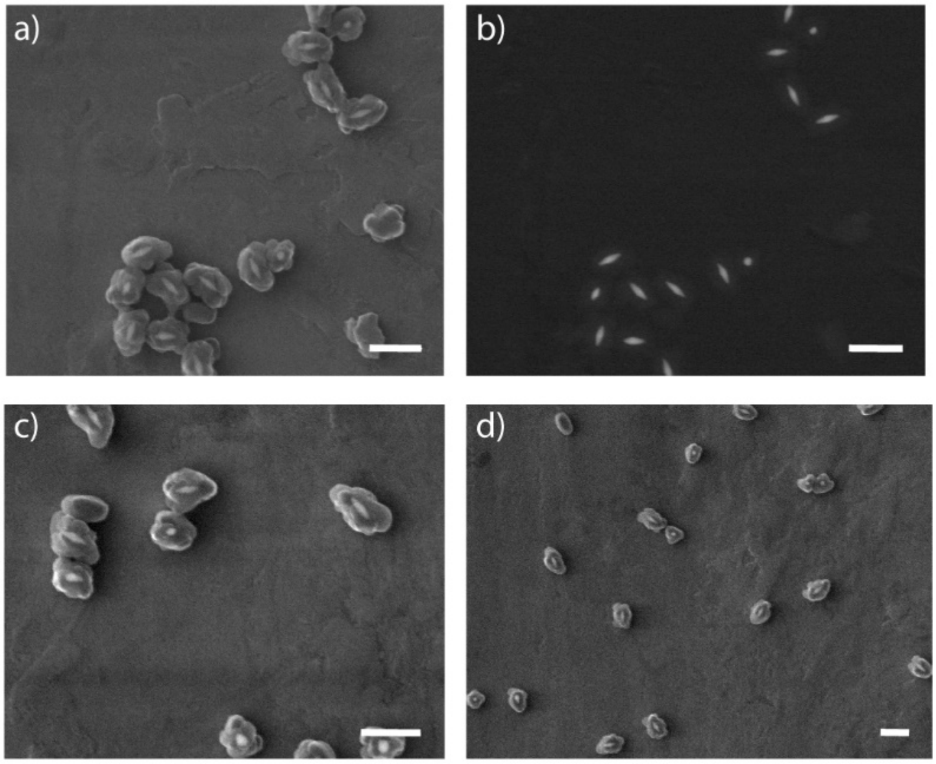

Figure S6: SEM images of the NU-1000 NCs placed in water before the PEG coating for a-b) 7 days, and after the PEG coating for c-d) 7 days.

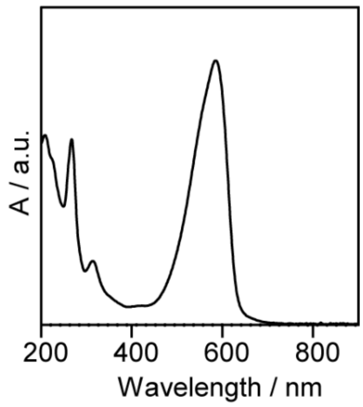

Figure S7: UV-Vis spectra of Cresyl Violet

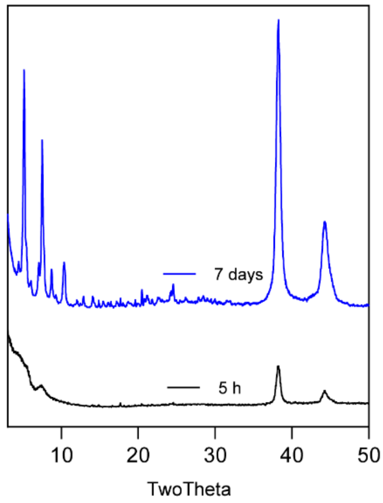

Figure S8: PXRD patterns of AuBy@NU-1000(CV)@PEG after 7 days in water (Blue) and AuBy@NU-1000 after 5 hours in water.

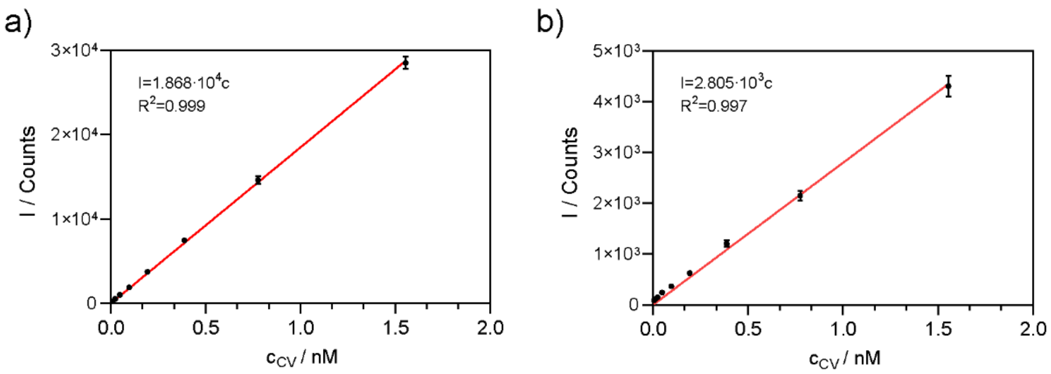

Figure S9: Calibration curves of a) CV in MeOH and b) CV in water**.**

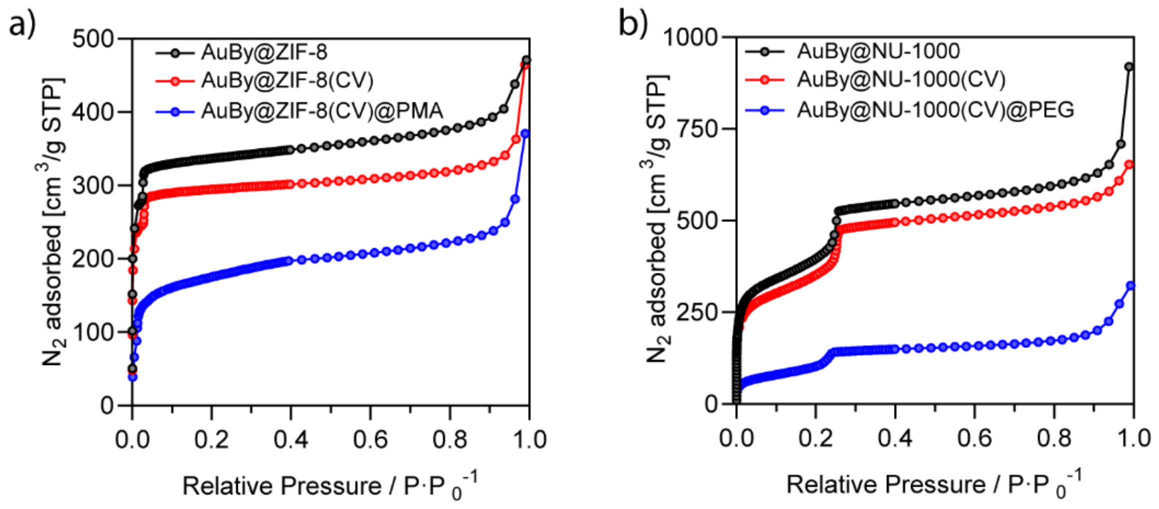

Figure S10: N_2 adsorption isotherms of a) ZIF-8 NCs and b) NU-1000 NCs

Table S1: S_{BET} values for ZIF-8 NCs

Sample	S_{BET} [$m^2 \cdot g^{-1}$]
AuBy@ZIF-8	1218
AuBy@ZIF-8(CV)	1086
AuBy@ZIF-8(CV)@PMA	650

Table S2: S_{BET} values for NU-1000 NCs

Sample	S_{BET} [$m^2 \cdot g^{-1}$]
AuBy@NU-1000	1424
AuBy@NU-1000(CV)	1274
AuBy@NU-1000(CV)@PEG	409

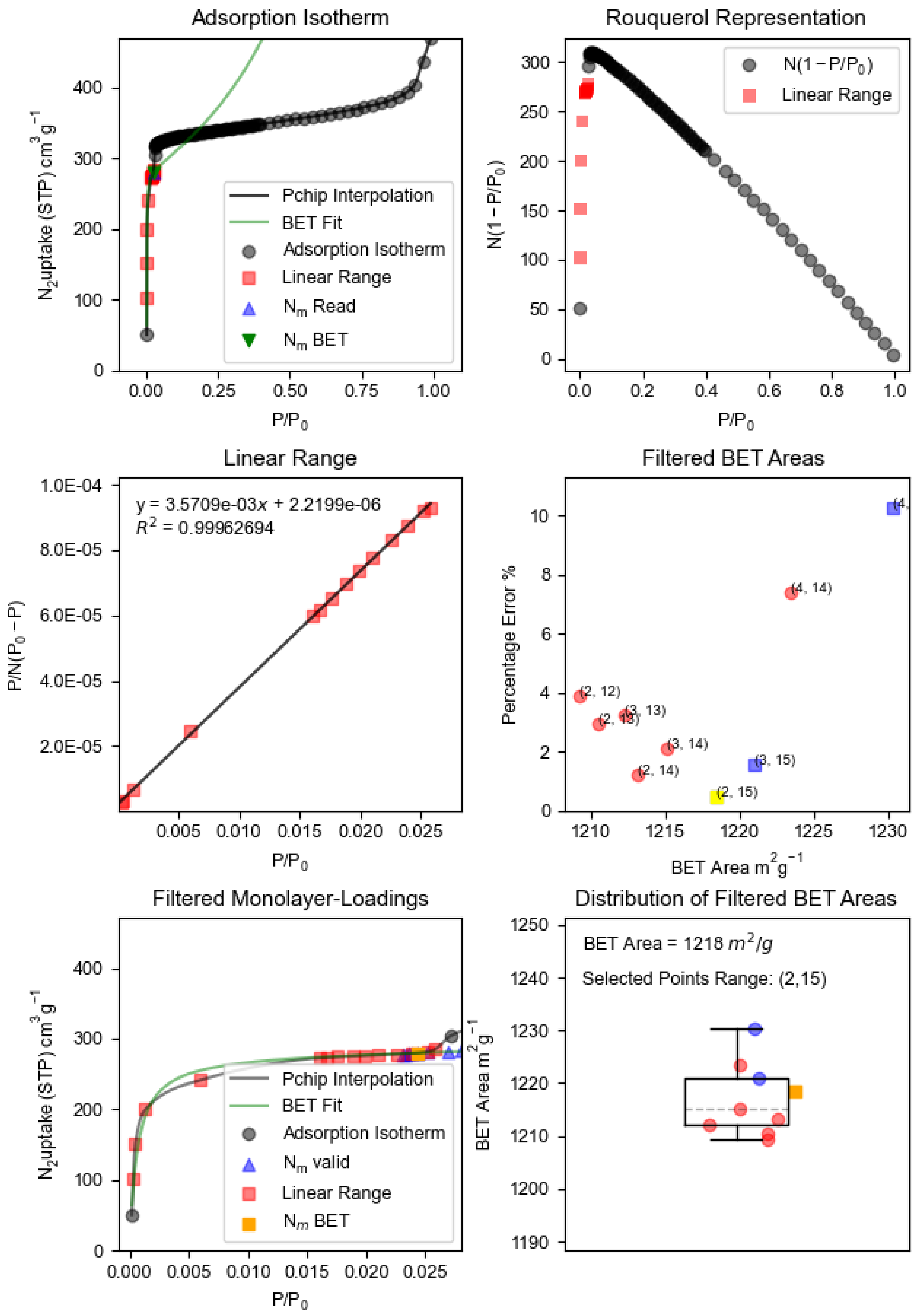

Figure S11: BETSI analysis for AuBy@ZIF-8

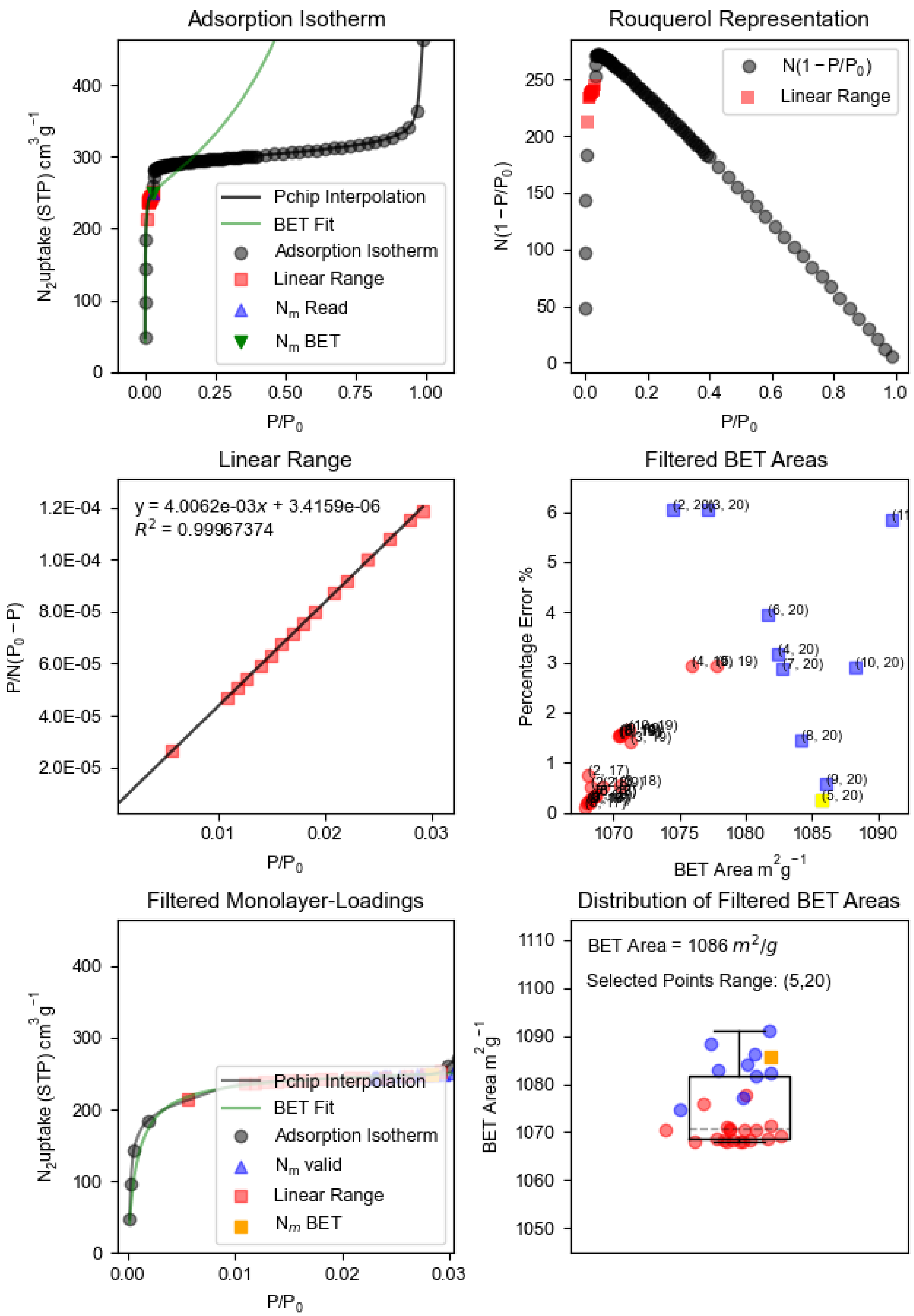

Figure S12: BETSI analysis for AuBy@ZIF-8(CV)

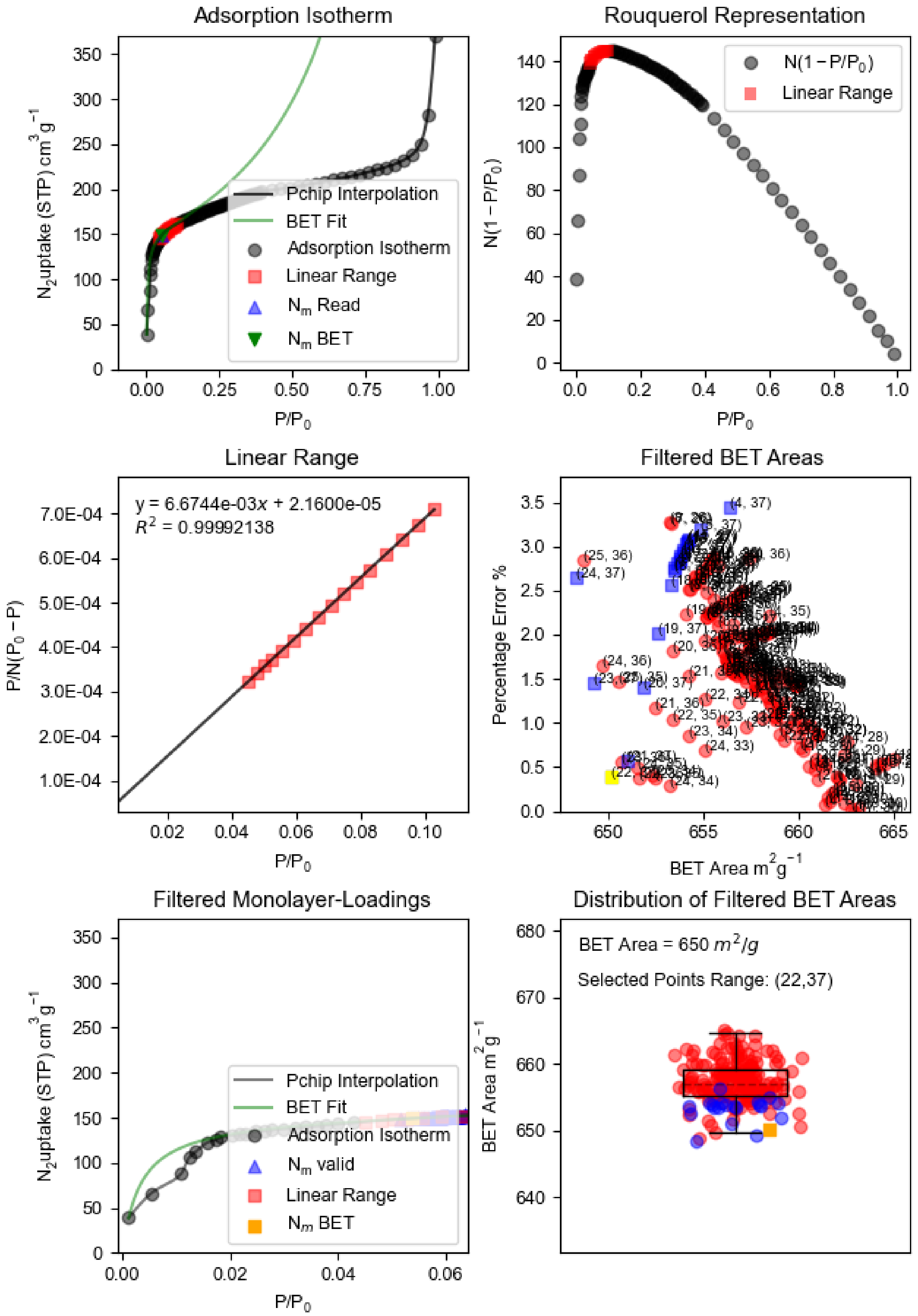

Figure S13: BETSI analysis for AuBy@ZIF-8(CV)@PMA

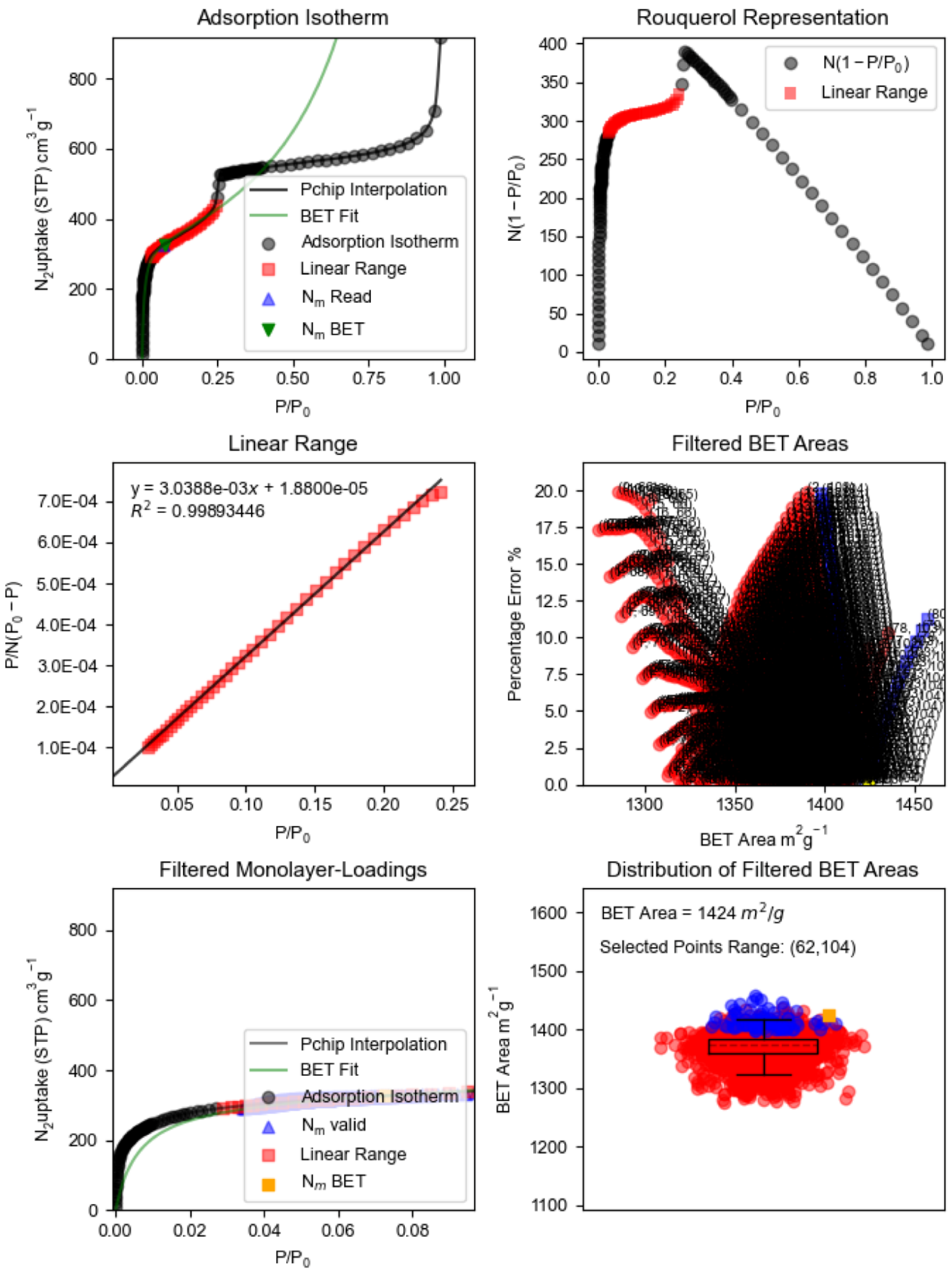

Figure S14: BETSI analysis for AuBy@NU-1000

u

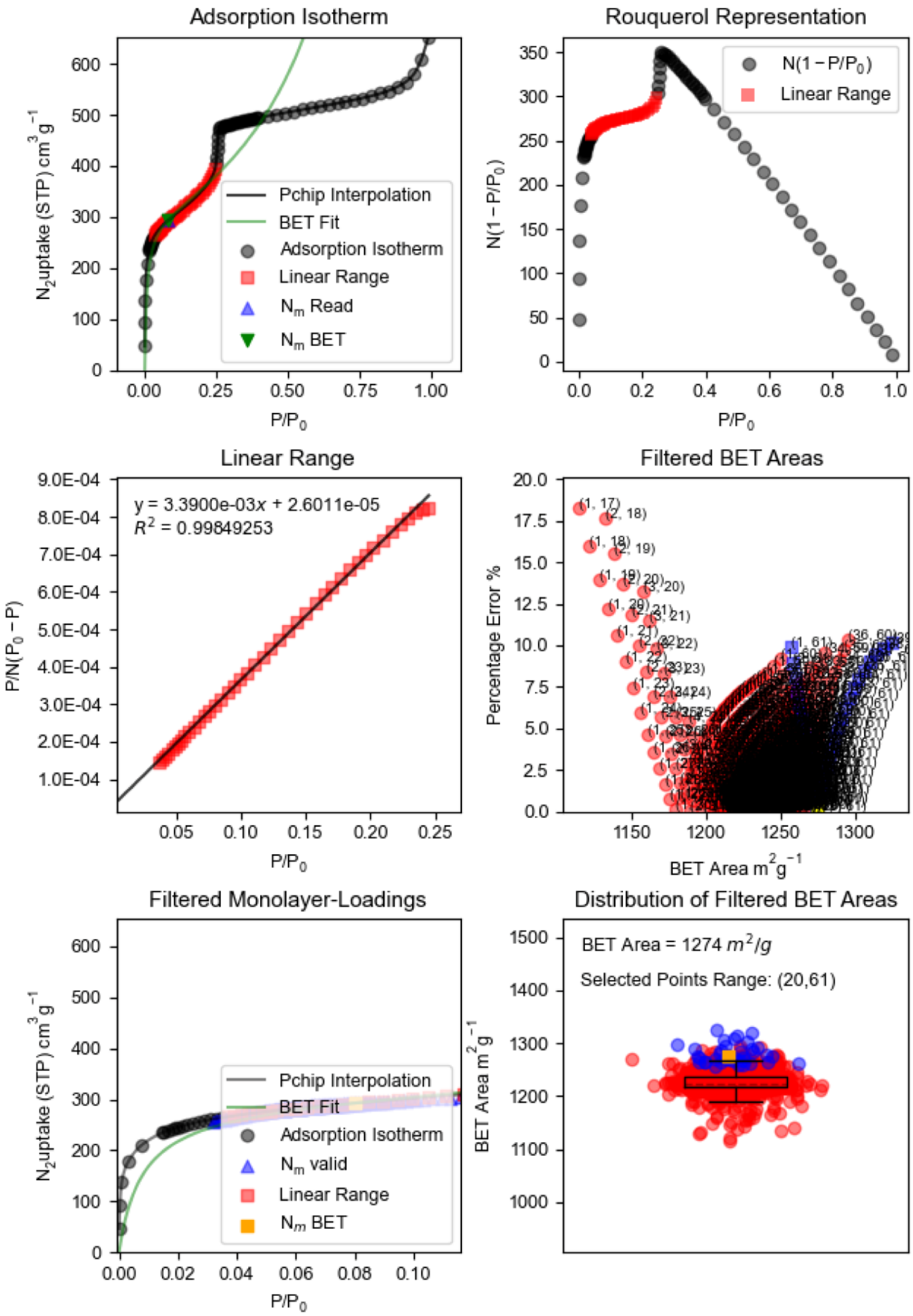

Figure S15: BETSI analysis for AuBy@NU-1000(CV)

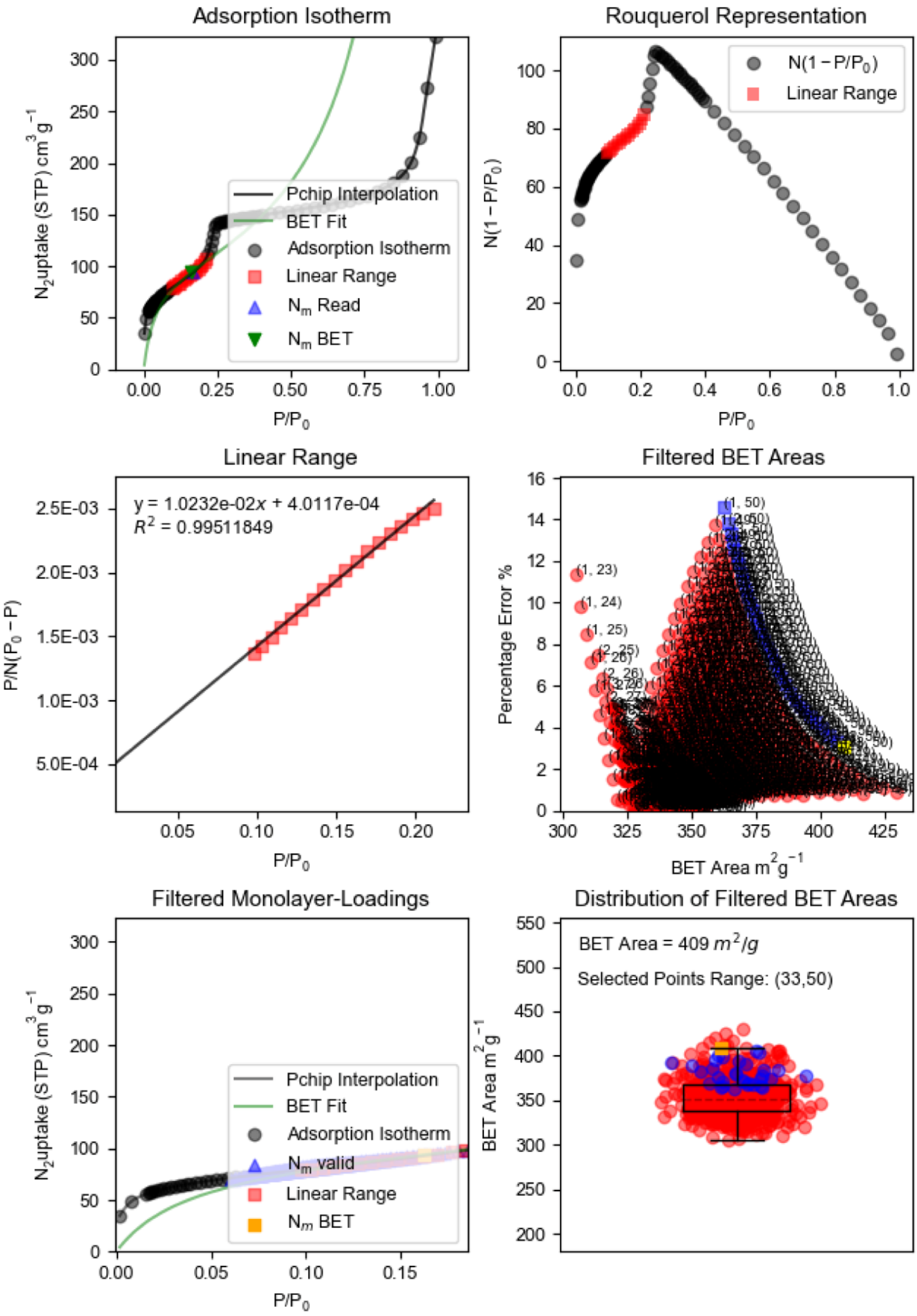

Figure S16: BETSI analysis for AuBy@NU-1000(CV)@PMA

Table S3: DLS and Zeta potential values for ZIF-8 and NU-1000 NCs

Sample	N ± sd	I ± sd	PDI ± sd	Z-Pot (mV)
AuBy@ZIF-8	158 ± 3	187 ± 4	0.14 ± 0.02	33.3 ± 1.1
AuBy@ZIF-8(CV)	166 ± 10	216 ± 8	0.12 ± 0.01	16.7 ± 1.0
AuBy@ZIF-8(CV)@PMA	185 ± 3	208 ± 3	0.05 ± 0.02	-25.0 ± 1.5
AuBy@NU-1000	165 ± 10	195 ± 12	0.20 ± 0.05	31.3 ± 0.9
AuBy@ NU-1000(CV)	174 ± 4	199 ± 14	0.18 ± 0.09	26.1 ± 2.0
AuBy@ NU-1000(CV)@PMA	181 ± 8	209 ± 20	0.11 ± 0.04	9.9 ± 1.2

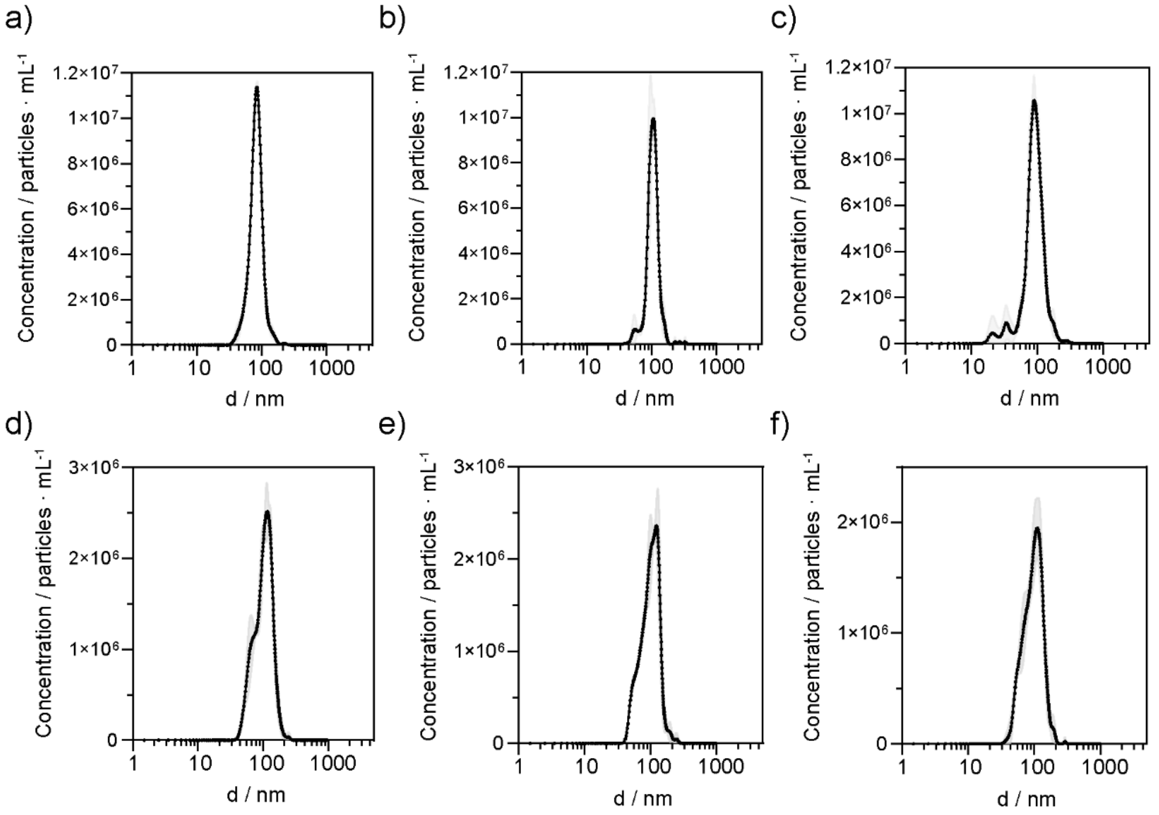

Figure S17: NTA measurements for a-c) ZIF-8 NCs (a. AuBy@ZIF-8, b. AuBy@ZIF-8(CV), c. AuBy@ZIF-8(CV)@PMA), and d-e) NU-1000 NCs (d. AuBy@NU-1000, e) AuBy@NU-1000(CV) and f) AuBy@NU-1000(CV)@PEG).

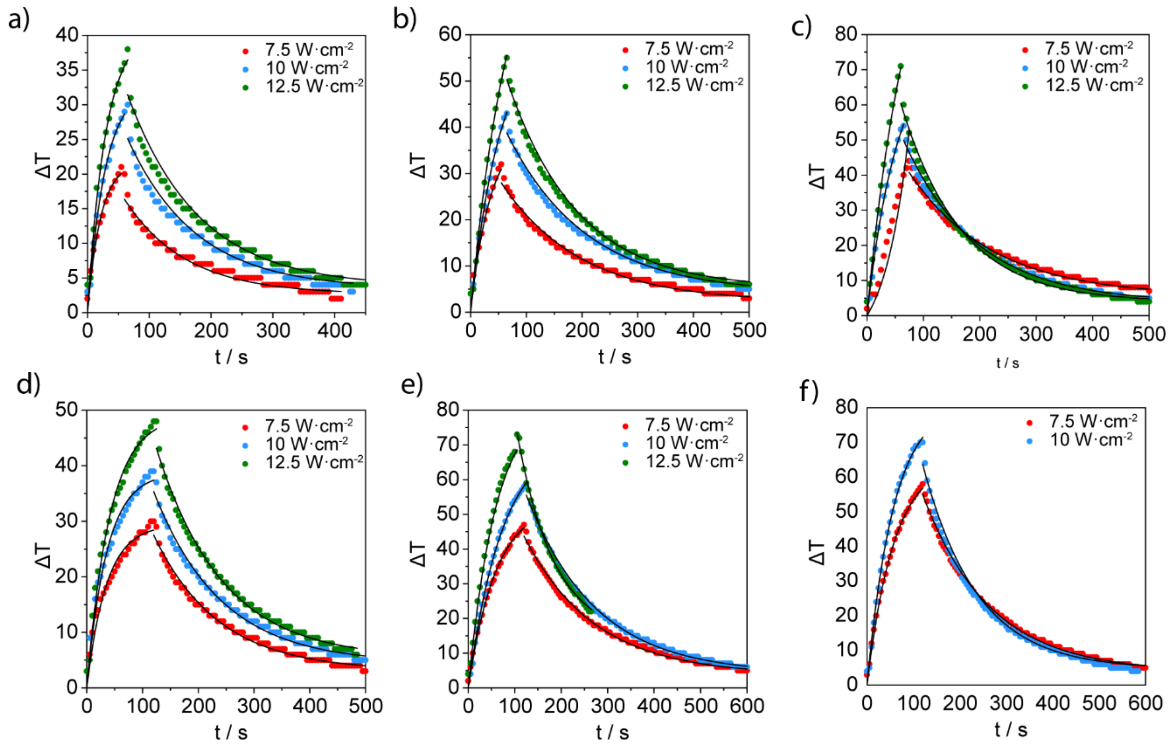

Figure S18: AuBy@ZIF-8@PEG heating curves in water for 1 minute irradiation at a) 50 pM, b) 100 pM, and 200 pM; and 2 minutes irradiation at d) 50 pM, e) 100 pM and f) 200 pM.

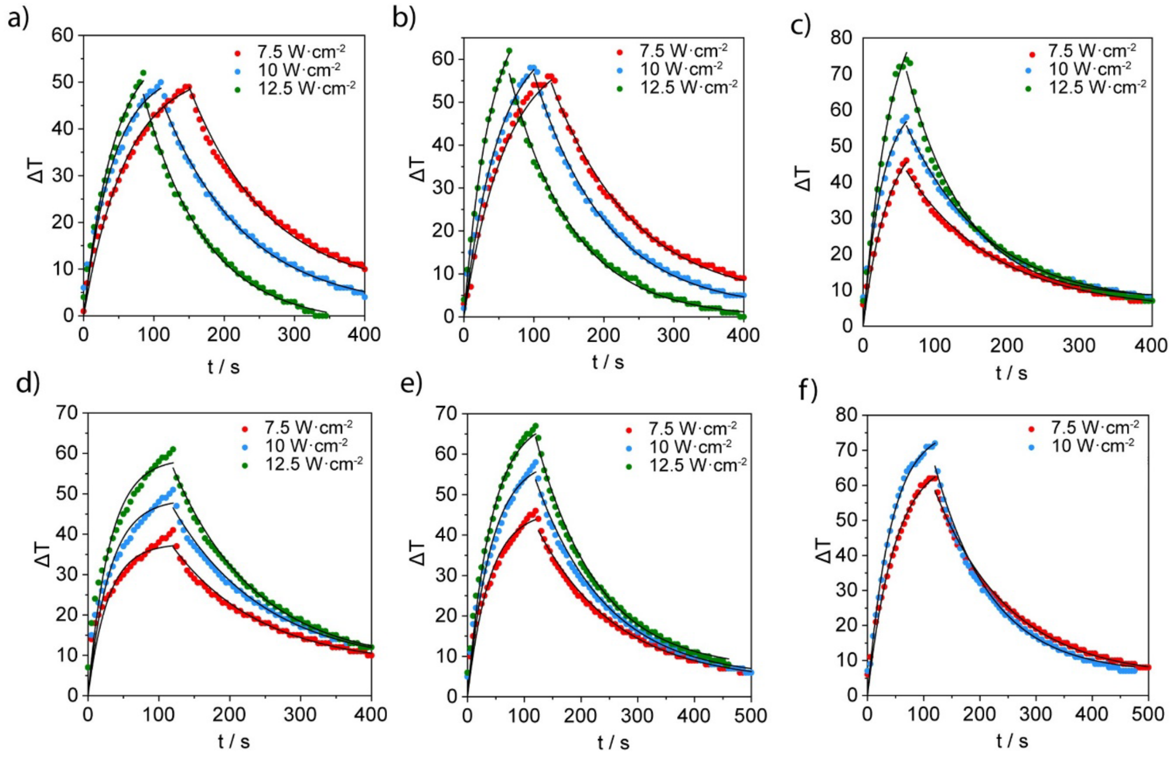

Figure S19: AuBy@NU-1000@PEG heating curves in water for 1 minute irradiation at a) 50 pM, b) 100 pM, and 200 pM; and 2 minutes irradiation at d) 50 pM, e) 100 pM and f) 200 pM.

Table S4: percentages of the CV released with respect to the encapsulated value inside the AuBy@ZIF-8(CV)@PMA at a concentration of 100 pM.

CV RELEASED		
W·cm^{-2}	%	FI Fold increase
Ctrl	11.07 ± 0.30	1.00 ± 0.03
7.5	22.89 ± 1.27	2.07 ± 0.11
10	26.04 ± 0.86	2.35 ± 0.08
12.5	35.06 ± 1.13	3.17 ± 0.10

Table S5: percentages of the CV released with respect to the encapsulated value inside the AuBy@NU-1000(CV)@PEG at a concentration of 100 pM.

	CV RELEASED	
W·cm^{-2}	%	FI Fold increase
Ctrl	5.21 ± 0.50	1.00 ± 0.10
7.5	23.31 ± 1.83	4.48 ± 0.35
10	27.19 ± 1.35	5.28 ± 0.28
12.5	30.18 ± 1.21	6.16 ± 0.43

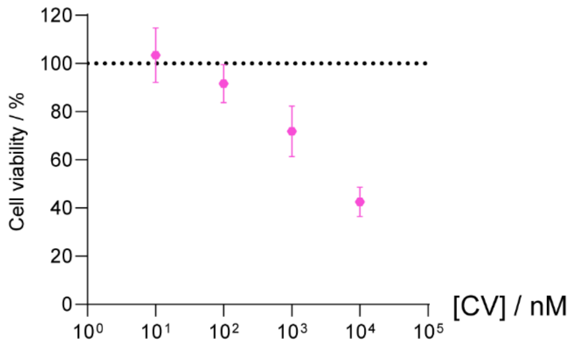

Figure S20. Cell viability of A459 cells incubated with CV for 24 h assessed by MTT assay.

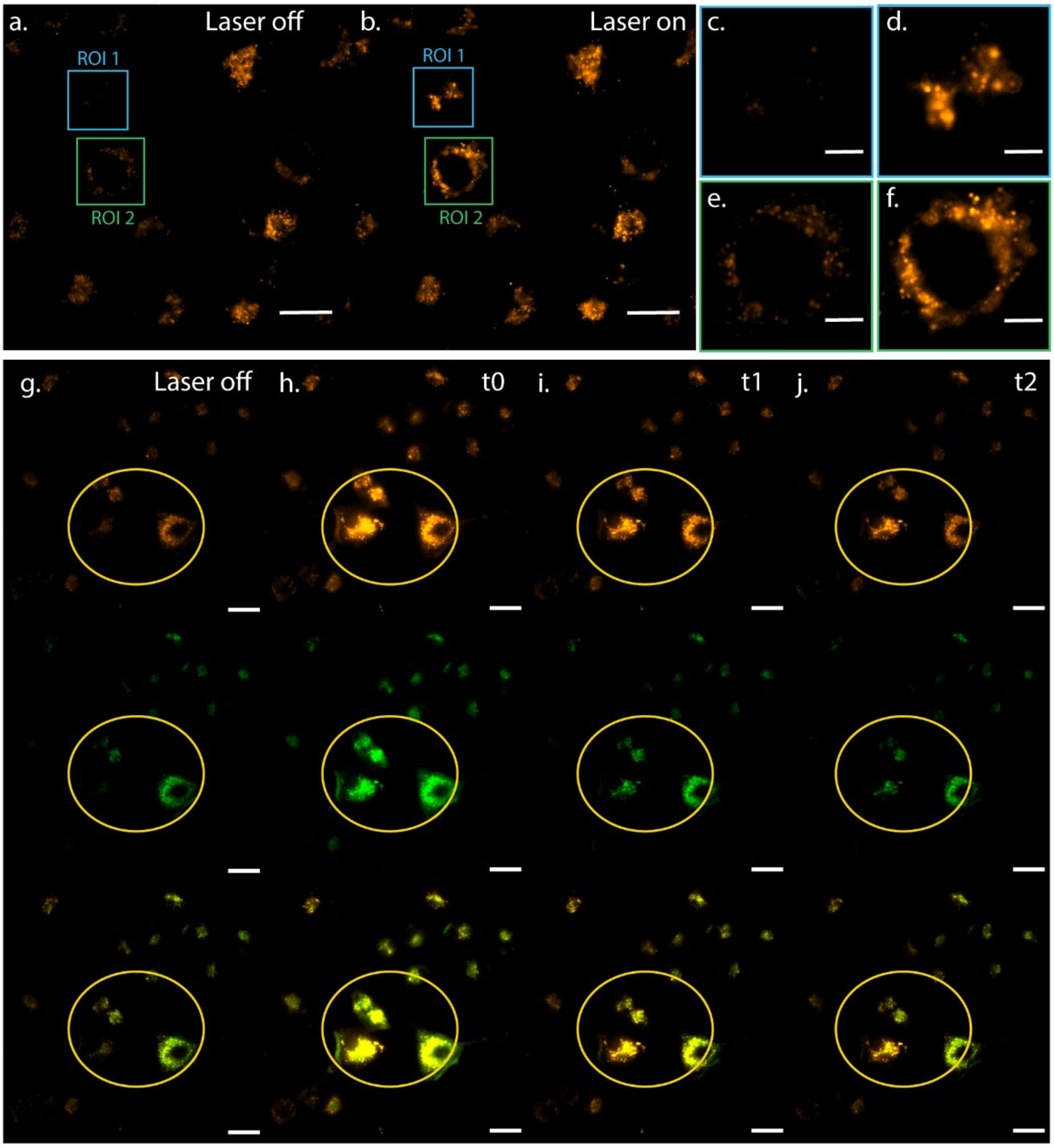

Figure S21. a) Microscopy images of the AuBy@ZIF-8(CV)@PMA before NIR laser irradiation b) after NIR laser irradiation of selected areas. Specific regions of interest (ROIs): c) ROI 1 and e) ROI 2 before irradiation and d) ROI 1 and f) ROI 2 after irradiation. g) Microscopy images show the fluorescence signal before NIR laser irradiation of the selected ROI, and sequential images at h) t0 = 10 s, i) t1 = 1 min, and j) t2 = 5 min after irradiation, highlighting the dynamic release of CV. The images feature the fluorescence signals from CV (orange), the fluorescence signal from Fluorescein (green), and the overlay of the two fluorescence channels. Scale bars 25 µm. Samples were incubated for 6 h at 50 pM.

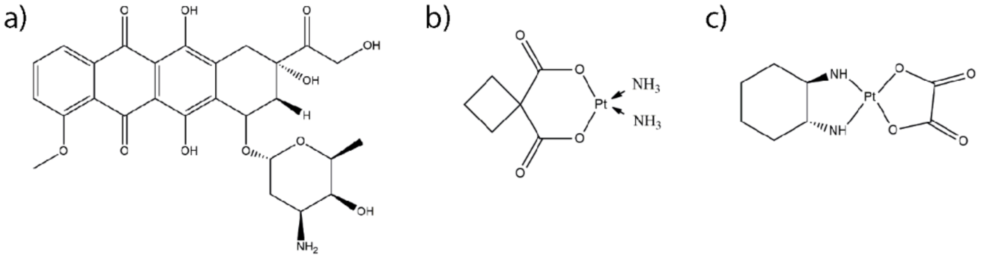

Figure S22: Drug molecules selected to perform the loading.

Table S6: DLS values for the drug-loaded NU-1000 NCs

Sample	N ± sd	I ± sd	PDI ± sd
AuBy@NU-1000(DOX)@PEG	161 ± 4	187 ± 4	0.18 ± 0.10
AuBy@NU-1000(CbPt)@PEG	167 ± 8	180 ± 6	0..20 ± 0.11
AuBy@NU-1000(OXA)@PEG	171 ± 5	182 ± 9	0.11 ± 0.07

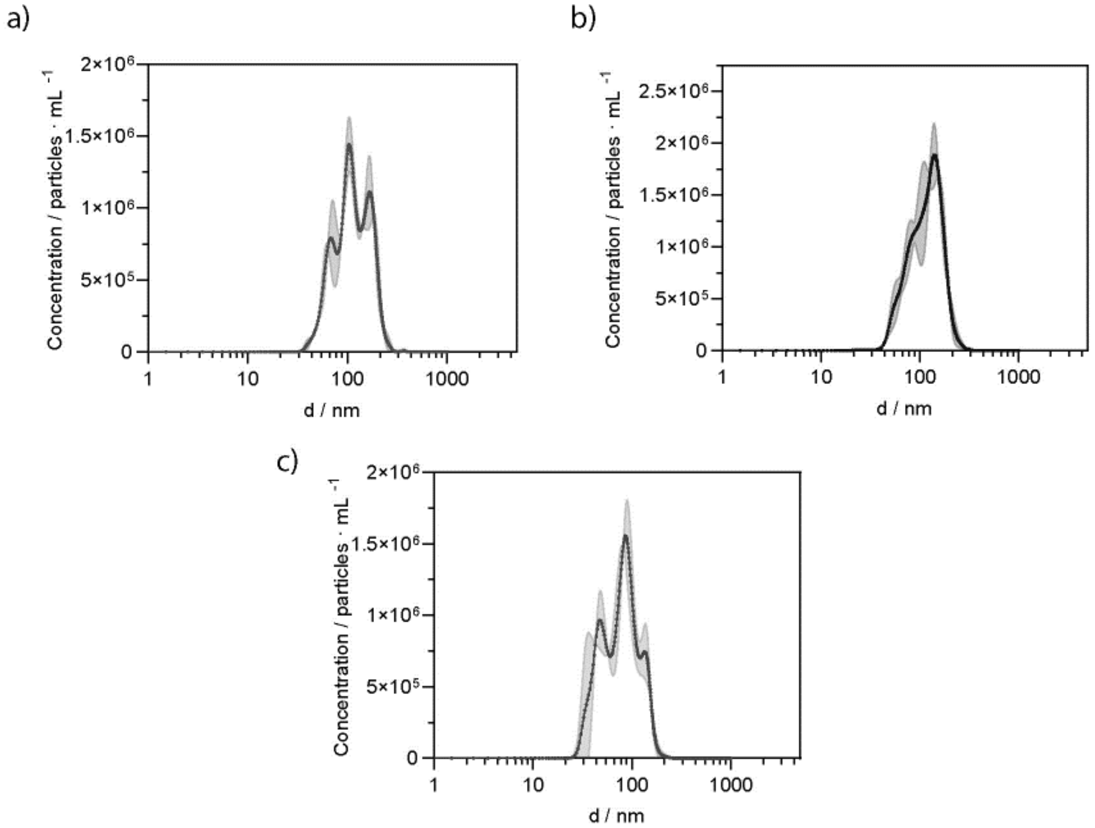

Figure S23: NTA measurements of a) AuBy@NU-1000(DOX)@PEG, b) AuBy@NU-1000(CbPt)@PEG and c) AuBy@NU-1000(OXA)@PEG

Table S7: Concentration values measured with NTA and ICP

	nM	part/mL	mg/mL Zr	mg/mL Au	mg/mL Pt	mM drug
AuBy@NU-1000(DOX)@PEG	10.63	6.40E+12	0.37	1.24	-	5.21
AuBy@NU-1000(CbPt)@PEG	8.04	4.84E+12	0.8	2.34	0.37	4.68
AuBy@NU-1000(OXA)@PEG	9.96	6.00E+12	0.73	2.16	0.31	3.94

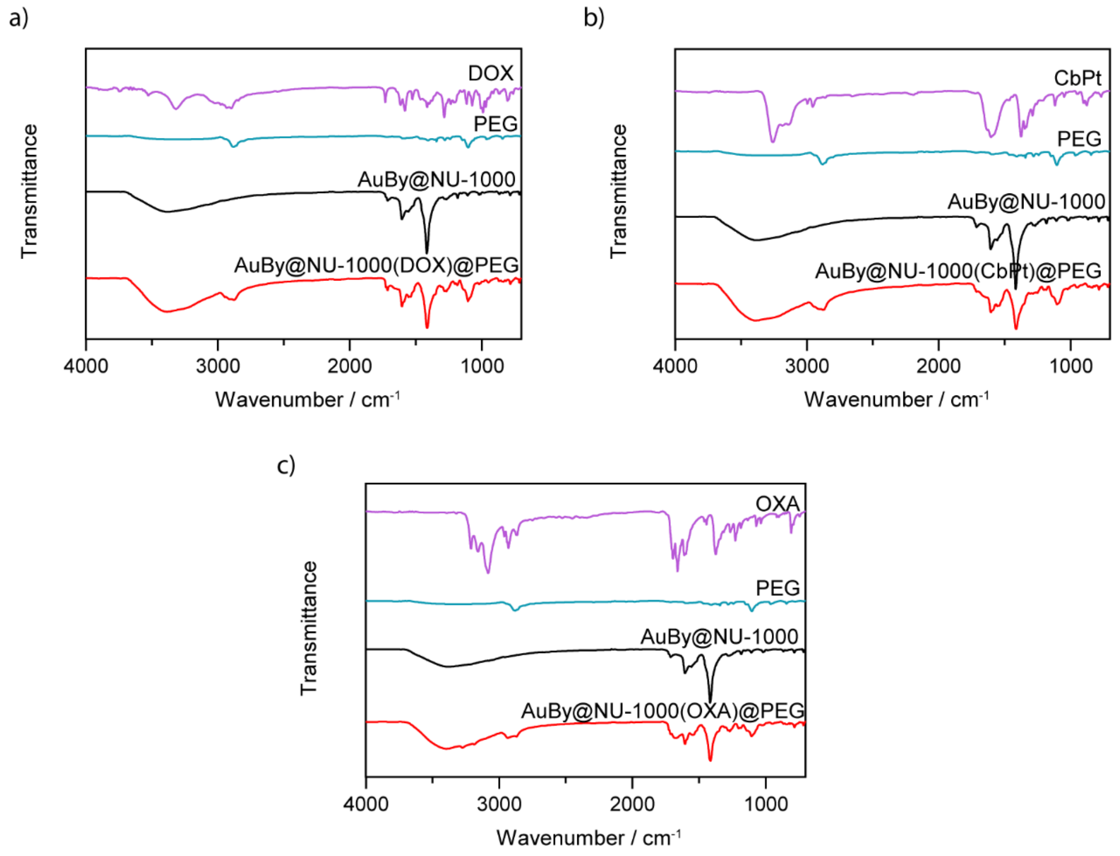

Figure S24: FTIR spectra for a) DOX loaded NCs, b) CbPt loaded NCs and c) OXA loaded NCs.

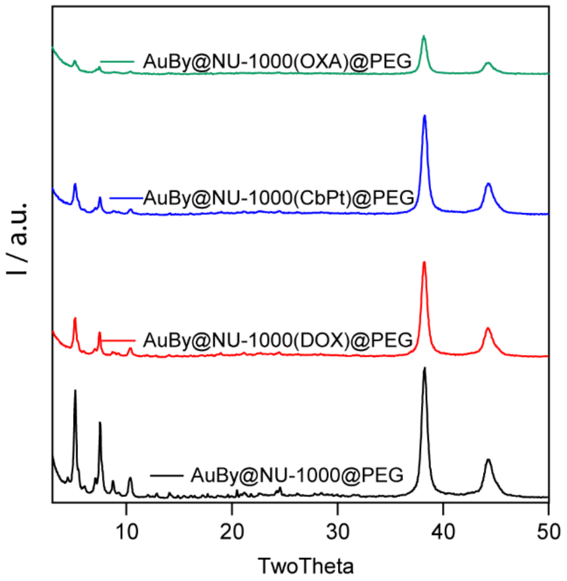

Figure S25: PXRD Crystallographic characterization of the samples after the drug loading and PEGylation.

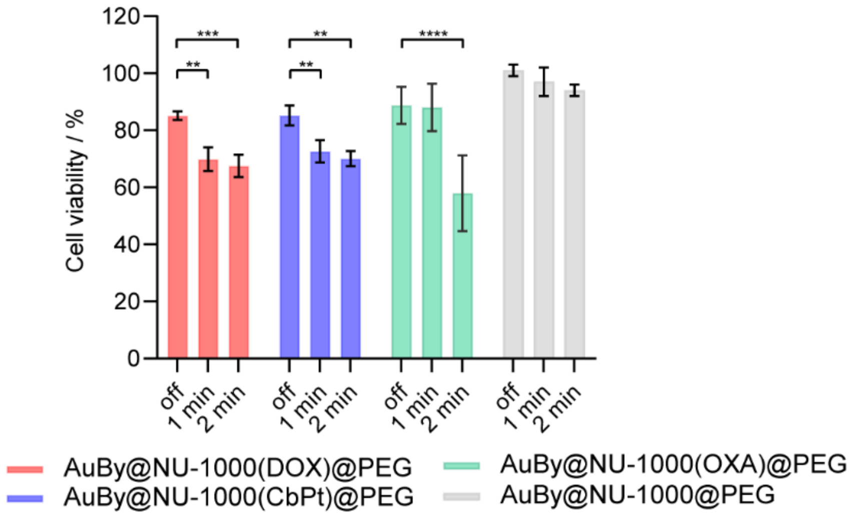

Figure S26: Cell viability of A549 cells incubated for 6 h with the loaded NCs using MTT assay 24 h after the irradiation at a 20 W·cm^{-2} irradiation power (concentration: 100 pM). Statistical analysis was assessed by two-way ANOVA test (**p = 0.0016, (***p = 0.0003 ****p < 0.0001).

Table S8: % of Cell viability 24h after NIR irradiation for the DOX loaded NCs

	AuBpy@NU-1000(DOX)@PEG		
t irr \ power	15 W·cm^{-2}	20 W·cm^{-2}	Ctrl AuBy@NU-1000@PEG
Laser off	85.22 ± 5.31	83.43 ± 4.29	100.98 ± 2.10
1 min	69.89 ± 4.12	79.15 ± 6.37	95.01 ± 3.12
2 min	67.55 ± 3.93	64.44 ± 7.55	91.92 ± 1.97

Table S9: % of Cell viability 24h after NIR irradiation for the CbPt loaded NCs

	AuBpy@NU-1000(CbPt)@PEG		
t irr \ power	15 W·cm^{-2}	20 W·cm^{-2}	Ctrl AuBy@NU-1000@PEG
Laser off	85.27 ± 3.49	85.27 ± 3.49	100.98 ± 2.10
1 min	72.65 ± 3.94	70.67 ± 1.85	95.01 ± 3.12
2 min	70.08 ± 2.63	62.52 ± 2.55	91.92 ± 1.97

Table S10: % of Cell viability 24h after NIR irradiation for the OXA loaded NCs

	AuBpy@NU-1000(OXA)@PEG		
t irr \ power	15 W·cm^{-2}	20 W·cm^{-2}	Ctrl AuBy@NU-1000@PEG
Laser off	92.03 ± 5.31	83.40 ± 4.19	100.98 ± 2.10
1 min	88.06 ± 8.29	61.62 ± 11.93	95.01 ± 3.12
2 min	57.92 ± 13.28	60.70 ± 6.51	91.92 ± 1.97

Table S11: selected irradiation condition for the 3D cell culture experiments

	t (s)	Irr power ($W \cdot cm^{-2}$)	Repetitions
Condition 1	60	20	2
Condition 2	30	30	2

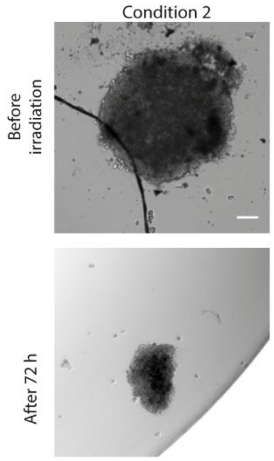

Figure S27: AuBy@NU-1000(CbPt)@PEG loaded **s**pheroids before and 72h after the irradiation.

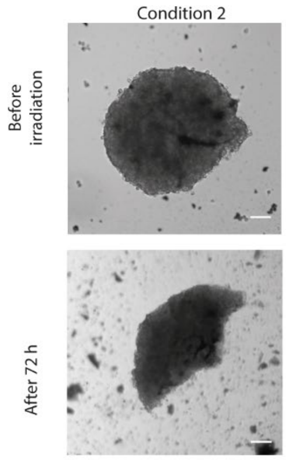

Figure S28: AuBy@NU-1000(OXA)@PEG loaded **s**pheroids before and 72h after the irradiation. Scale bar 100 µm.

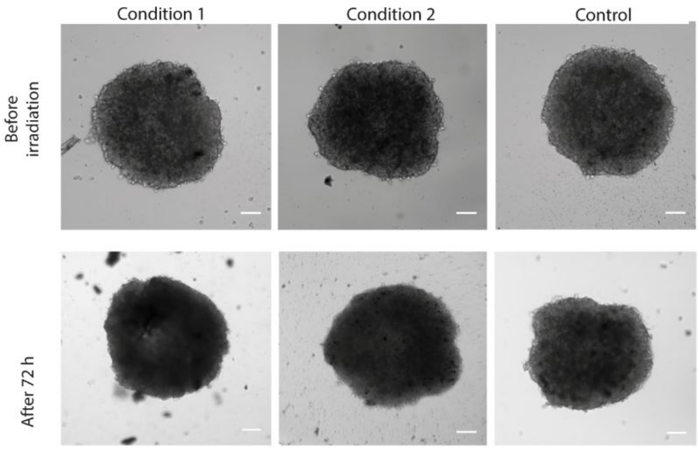

Figure S29: AuBy@NU-1000@PEG loaded **s**pheroids before and 72h after the irradiation. Scale bar 100 µm.

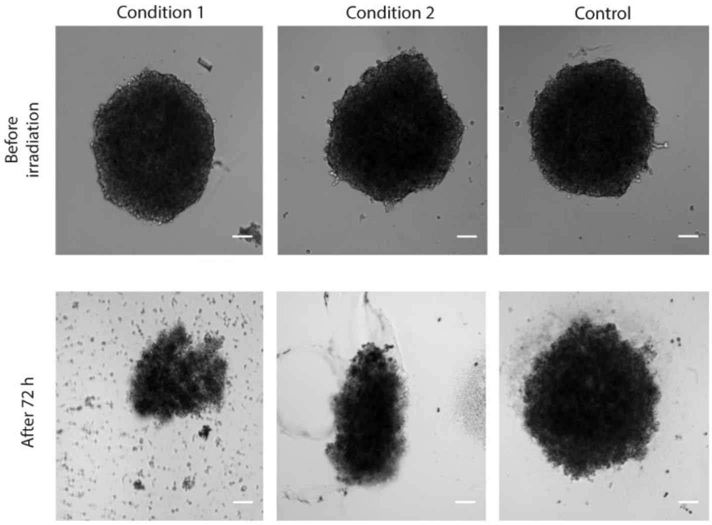

Figure S30: Free CbPt incubated with **s**pheroids before and 72h after the irradiation. Scale bar 100 µm.

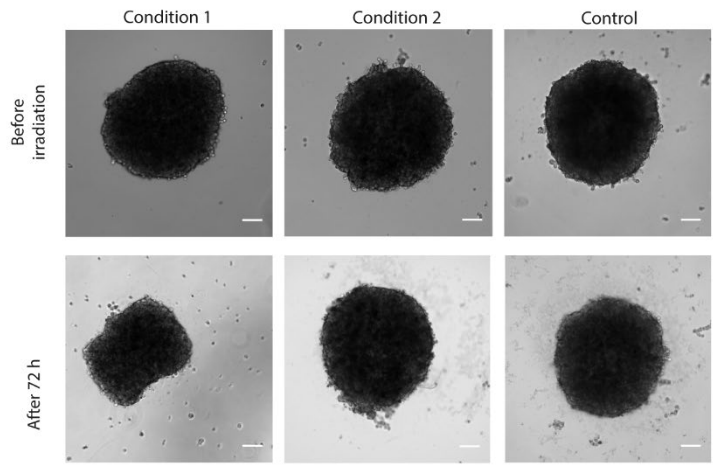

Figure S31: Free OXA incubated with **s**pheroids before and 72h after the irradiation. Scale bar 100 µm.

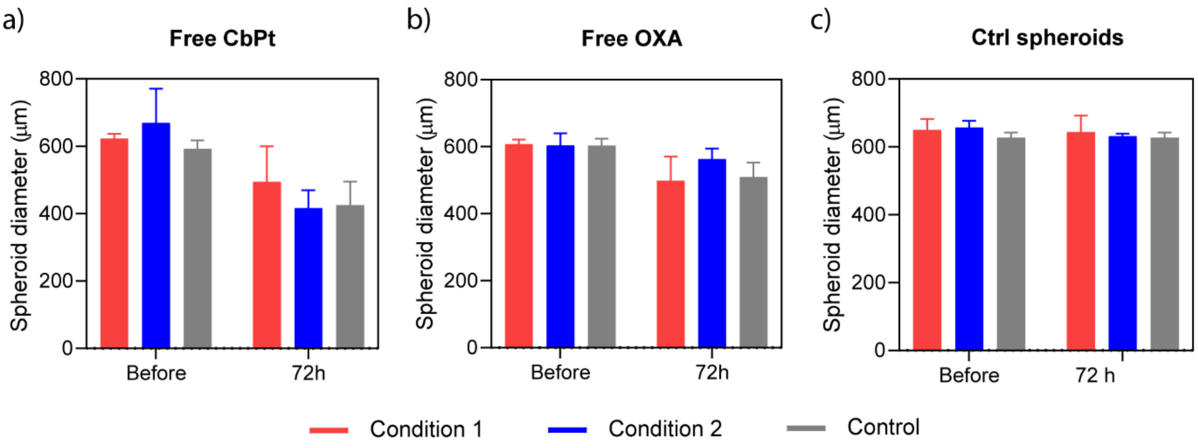

Figure S32: Spheroids diameter analysed from microscopy images before and 72h after the irradiation for a) Free CbPt, b) Free OXA and c) Control spheroids. Experiments were carried out using n = 3. Error bars represent standard deviation. Statistical analysis was assessed by two-way ANOVA test showing no significant difference among the samples.

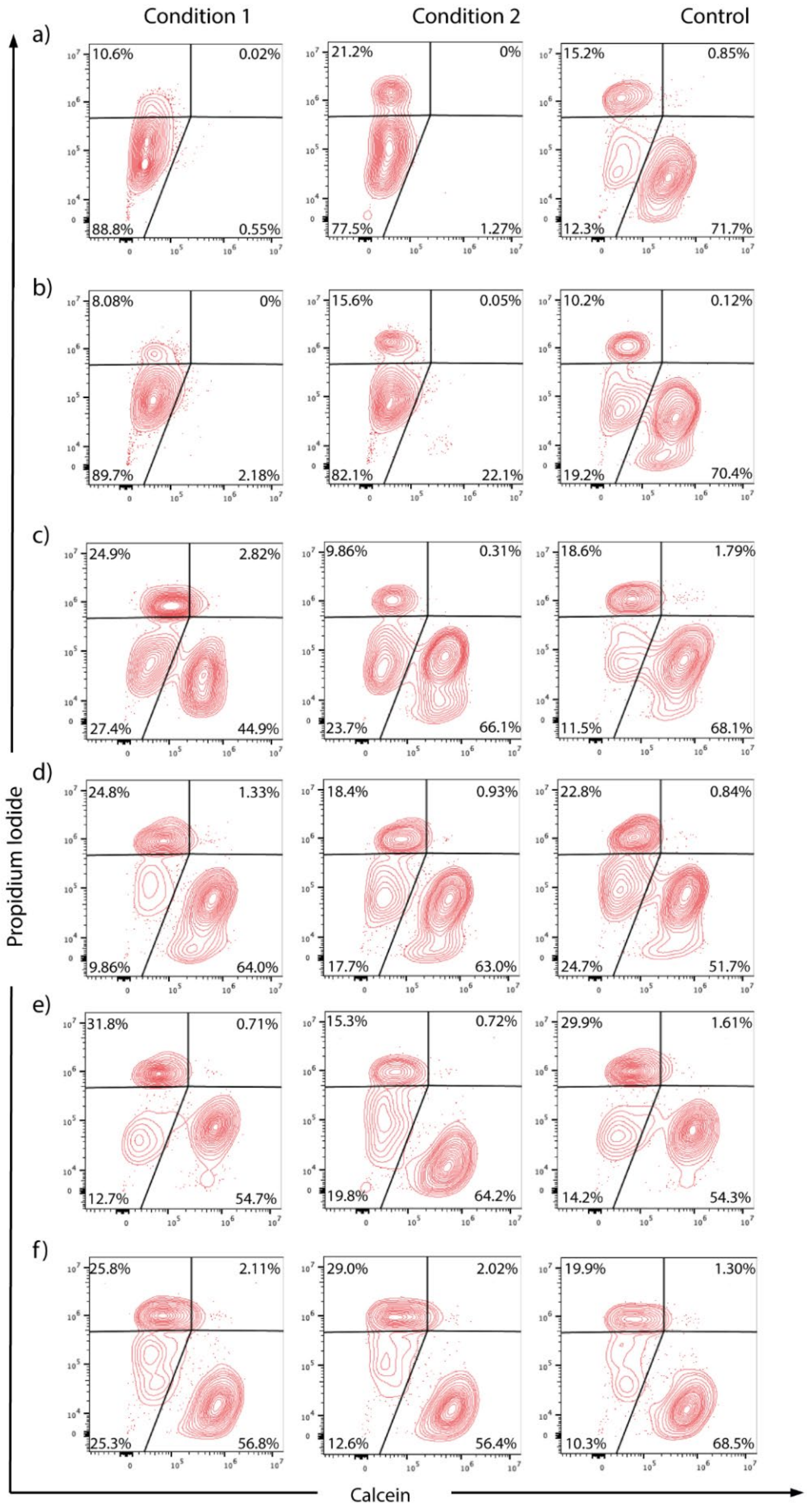

Figure S33: Representative flow cytometric dot-plots showing Calcein and PI-positive cells 24 h after NIR irradiation of the spheroids for a) AuBy@NU-1000(CbPt)@PEG, b) AuBy@NU-1000(OXA)@PEG, c) AuBy@NU-1000@PEG, d) Free CbPt, e) Free OXA, f) Control spheroids.

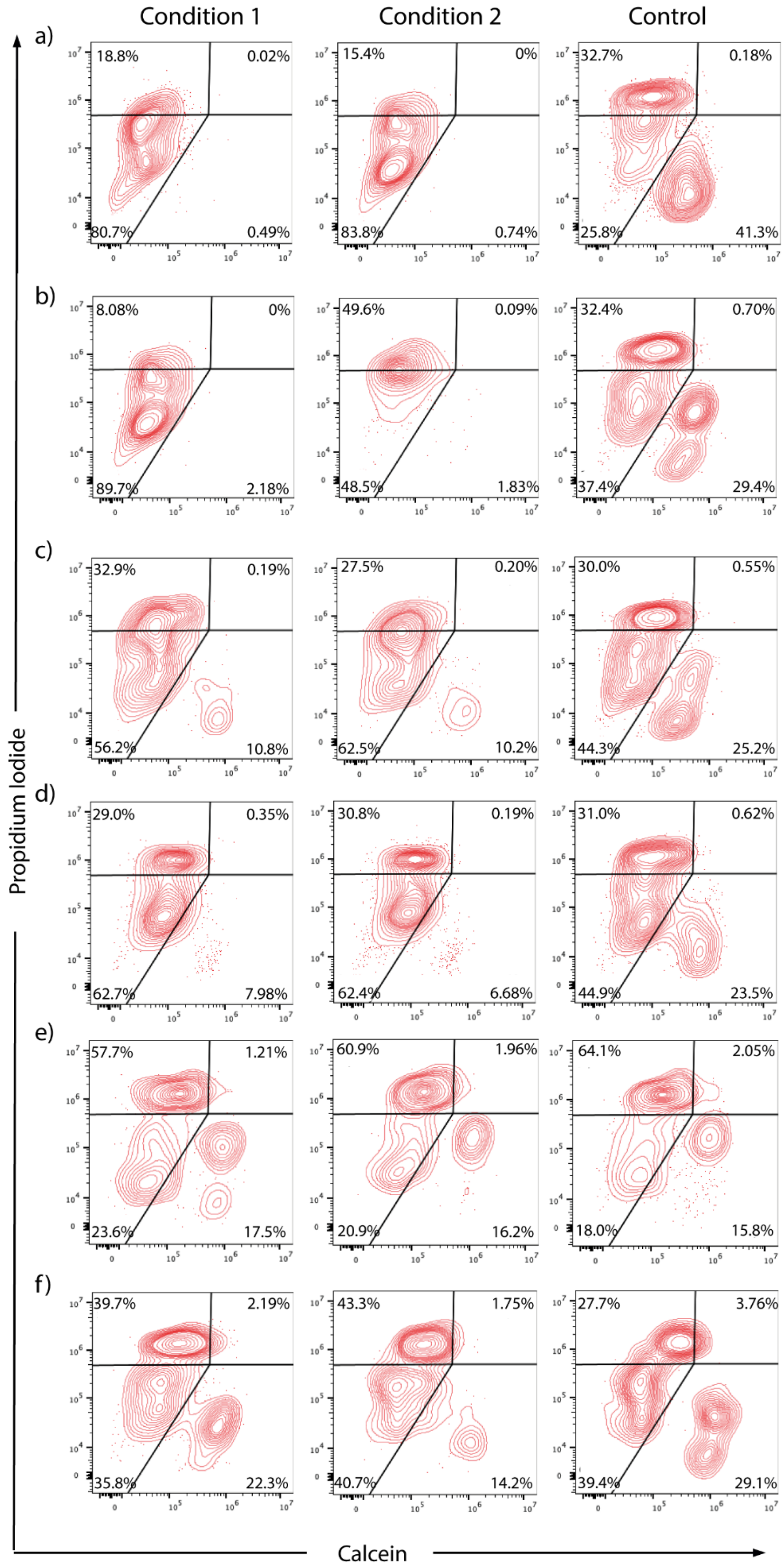

Figure S34: Representative flow cytometric dot-plots showing Calcein and PI-positive cells 72 h after NIR irradiation of the spheroids for a) AuBy@NU-1000(CbPt)@PEG, b) AuBy@NU-1000(OXA)@PEG, c) AuBy@NU-1000@PEG, d) Free CbPt, e) Free OXA, f) Control spheroids.

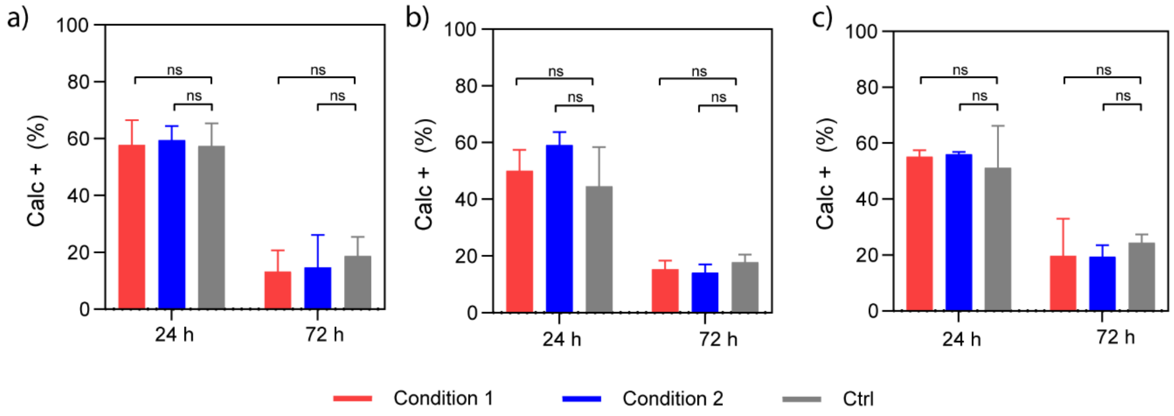

Figure S35: Flow cytometry results of % of calcein stained cells after 24h and 72h of irradiation for a) Free CbPt, b) Free OXA and c) control spheroids.
